# Phosphorylcholine-Based Contact Lenses for Sustained Release of Resveratrol: Design, Antioxidant and Antimicrobial Performances, and *In Vivo* Behavior

**DOI:** 10.1021/acsami.2c18217

**Published:** 2022-12-10

**Authors:** Maria Vivero-Lopez, Ana F. Pereira-da-Mota, Gonzalo Carracedo, Fernando Huete-Toral, Ana Parga, Ana Otero, Angel Concheiro, Carmen Alvarez-Lorenzo

**Affiliations:** † Departamento de Farmacología, Farmacia y Tecnología Farmacéutica, I+D Farma (GI-1645), Facultad de Farmacia, Instituto de Materiales (iMATUS) and Health Research Institute of Santiago de Compostela (IDIS), Universidade de Santiago de Compostela, 15782Santiago de Compostela, Spain; ‡ Ocupharm Research Group, Faculty of Optics and Optometry, Complutense University of Madrid, C/Arcos del Jalon 118, 28037Madrid, Spain; § Department of Optometry and Vision, Faculty of Optics and Optometry, Complutense University of Madrid, C/Arcos del Jalon 118, 28037Madrid, Spain; ∥ Departamento de Microbiología y Parasitología, Facultad de Biología, Edificio CIBUS, Universidade de Santiago de Compostela, 15782Santiago de Compostela, Spain

**Keywords:** medicated contact lenses, resveratrol, free water, antioxidant, antimicrobial, *in vitro*−*in vivo* correlations, ocular tissue biodistribution

## Abstract

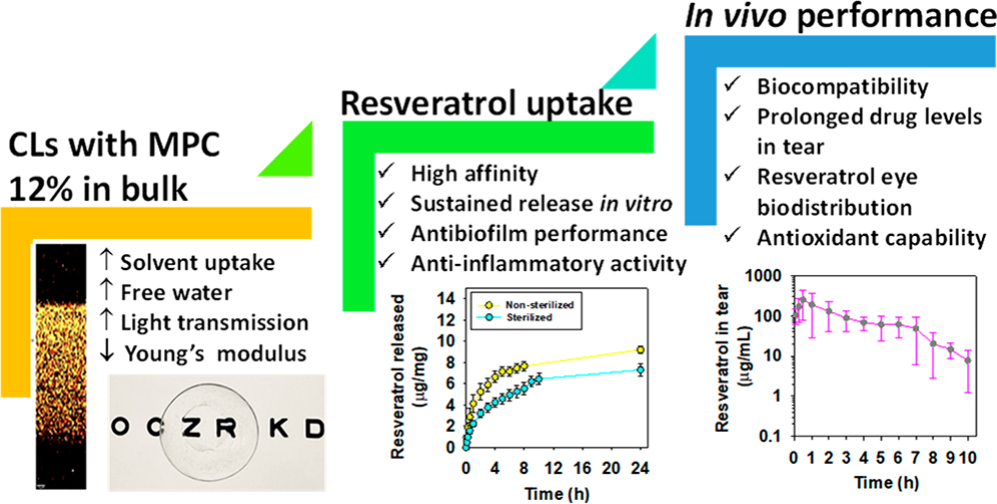

Design of advanced contact lenses (CLs) demands materials that are safe and comfortable for the wearers and that preserve the normal eye microbiota, avoiding chronic inflammation and biofilm development. This work aimed to combine the natural antibiofouling phosphorylcholine and the antioxidant and prebiotic resveratrol as integral components of CLs that may have the additional performance of preventing oxidative-stress related eye diseases. Different from previous uses of 2-methacryloyloxyethyl phosphorylcholine (MPC) as coating, we explored the feasibility of adding MPC at high proportions as a comonomer of 2-hydroxyethyl methacrylate (HEMA)-based hydrogels while still allowing for the loading of the hydrophobic resveratrol. Homogeneous distribution of MPC along the hydrogel depth (confirmed by Raman spectroscopy) notably increased solvent uptake and the proportion of free water while it decreased Young’s modulus. Relevantly, MPC did not hinder the uptake of resveratrol by CLs (>10 mg/g), which indeed showed network/water partition coefficients of >100. Protocols for CLs sterilization and loading of resveratrol under aseptic conditions were implemented, and the effects of tear proteins on resveratrol release rate were investigated. CLs sustained resveratrol release for more than 24 h *in vitro*, and sorption of albumin onto the hydrogel, although attenuated by MPC, slowed down the release. The combination of MPC and resveratrol reduced *P. aeruginosa* and *S. aureus* growth as tested in a novel hydrogel disk-agar interface biofilm growth setup. The developed CLs showed excellent anti-inflammatory properties and biocompatibility in *in ovo* and rabbit tests and provided higher and more prolonged levels of resveratrol in tear fluid, which favored resveratrol biodistribution in anterior and posterior eye segments compared to eye drops. Correlations between the release profiles of resveratrol *in vitro* and *in vivo* were assessed. Relevantly, the CLs preserved the antioxidant properties of resveratrol during the entire 8 h of wearing. In sum, CLs prepared with high proportion in MPC may help address safety and comfort requirements while having drug releasing capabilities.

## Introduction

More than 140 million people wear contact lenses (CLs) worldwide, and the number of wearers is expected to grow further due to planned improvements in CL material design that expand into diagnosis and therapy applications.^1−5^ CLs offer high visual acuity but the prolonged use causes discomfort due to changes in tear fluid dynamics and oxygen diffusion limitations and also increases the risk of microbial-related ocular diseases.^6^ The surface of CLs is prone to be colonized by bacteria from skin (fingers) that can form biofilms.^7,8^ Moreover, the presence of the CL on the ocular surface can damage the corneal epithelium and interferes with tearing and blinking, facilitating the development of microbial keratitis.^7,9^ Indeed, prolonged wearing of CLs has been demonstrated to alter the eye microbiota, which may weaken eye protection against infections.^10^

**Figure 1:**
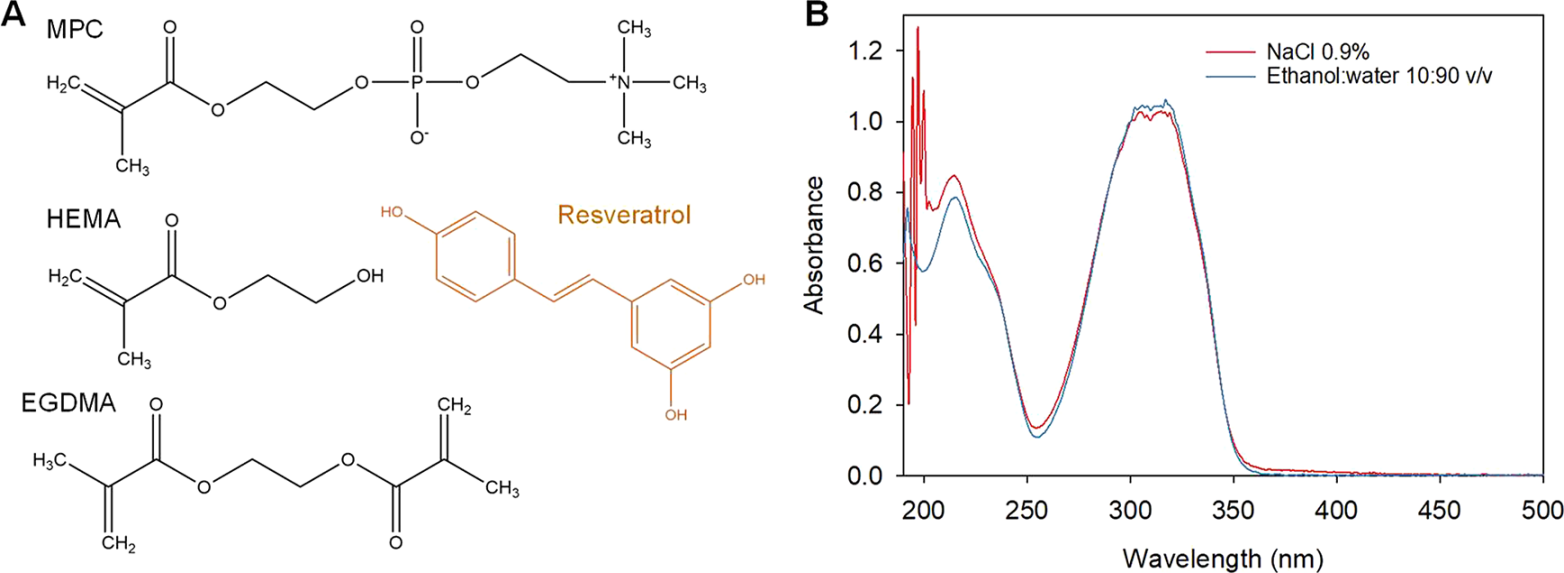
(A) Chemical structure of 2-methacryloyloxyethyl phosphorylcholine (MPC), 2-hydroxyethyl methacrylate (HEMA), ethylene glycol dimethacrylate (EGDMA), and *trans-*resveratrol and (B) UV–vis spectra of resveratrol solutions (8 μg/mL) prepared in the medium used for the loading of the hydrogels (ethanol/water 10:90 v/v) and in the medium used for the release tests (NaCl 0.9% aq solution).

Incorporation of antibiofouling components on the surface of CLs has been investigated.^6,8,11^ A variety of coatings containing antimicrobial and antioxidant drugs and inorganic ions and nanoparticles are being tested as a way of counteracting the risk of ocular infections.^12−14^ However, most of these approaches still involve many steps that hamper the industrial scale-up of the antibiofouling material and also raise concerns on the prophylactic use of wide-spectrum antimicrobial agents that may foster bacteria resistances. In the search of efficient but still secure strategies to increase CLs comfort and safety, bioinspired approaches are gaining raising attention.^15^ As an example, nanowrinkled surface patterns bioinspired in the zebrafish cornea have recently been demonstrated to prevent bacteria adhesion while still ensuring optical transparency.^16^ A more straightforward strategy is the use of monomers that resemble the zwitterionic functionalities of some lipids at the cell membranes. Compared to other zwitterionic groups such as carboxybetaine and sulfobetaine, derivatives of phosphorylcholine are more stable against pH changes in the physiological environment, more biocompatible, and more efficient in preventing cell adhesion.^17^ Indeed, surface treatment of CLs with 2-methacryloyloxyethyl phosphorylcholine (MPC) (Figure 1), either as grafted polymer or as a cross-linked layer, has been demonstrated to attenuate the deposition of biomacromolecules and cells owing to a combined effect of enhanced wettability and increased mobility of water and cells on the CLs surface.^18−20^

Compared to coatings which involve additional steps in the CLs manufacture, incorporation during the CLs polymerization of monomers resistant to bacterial attachment may be simpler.^21^ Currently, there is one commercially available soft CL that contains MPC (Proclear) and is claimed to be more comfortable because of its enhanced wettability.^22^ Proclear contains 3% MPC (equivalent to 101 mM) in a mixture with 2-hydroxyethyl methacrylate (HEMA).^23^ In a previous report, we observed that these CLs were less prone to *Pseudomonas aeruginosa* PAO1 biofilm formation, but no changes on *Staphylococcus aureus* biofilms were recorded compared to HEMA networks prepared in the absence of MPC.^24^ We also evidenced the difficulties of mixing MPC with silicone hydrogel components, which caused microphase separation.^24^

In parallel to the use of antibiofilm structural components, the incorporation of natural antioxidants that can prevent changes in microbiota and chronic inflammatory response of the eye to CL wearing is being explored.^25^ Resveratrol is a potent antioxidant agent that has shown adequate ocular tolerance, aids in the management of oxidative-stress related eye diseases, and favors the healing of corneal epithelial cells.^26^ Relevantly, resveratrol may act as prebiotic and has been reported to restore natural microbiota in several body tissues, including eye tissues.^27,28^ Moreover, resveratrol can inhibit Gram-positive and Gram-negative bacteria growth *in vitro* if used at high concentrations.^24,28,29^ However, when ingested with the diet, resveratrol absorption is constrained by its poor aqueous solubility which limits the bioavailability; it is considered as a class II molecule in the Biopharmaceutics Classification System.^30^ Thus, direct administration of resveratrol to eye tissues in topical formulations may notably improve the ocular health.^31−33^ Incorporation of resveratrol and other antioxidant agents into CLs for sustained release has recently been attempted,^24,25^ but optimization of their performances and *in vivo* evaluation are still missed.

In a previous study, we observed that HEMA-based hydrogels loaded lower amounts of resveratrol than silicone-based hydrogels, but HEMA-based hydrogels had the advantage of preventing irreversible binding and thus delivered the amount loaded.^24^ The aim of this work was to explore the possibility of preparing HEMA-based hydrogels with MPC proportions well beyond 101 mM and that can load resveratrol in order to combine antifouling, anti-inflammatory, and antioxidant properties. An increase in MPC is expected to enhance the wettability and amount of free water in the hydrogels contributing to the antifouling performance, but at the same time it may become a barrier for the loading of a hydrophobic therapeutic substance such as resveratrol. CLs with well-balanced hydrophilic/hydrophobic features may provide a sustained release of resveratrol on the cornea avoiding its premature clearance from the eye surface and facilitating resveratrol access to eye tissues for the management of ocular pathologies associated with oxidative stress. There is still a paucity of information on how *in vitro* release profiles from CLs may forecast the *in vivo* release patterns of therapeutic substances to the tear fluid and more relevantly the biodistribution in ocular tissues.^3^ Thus, an additional aim of the work was to gain an insight into the feasibility of using MPC-bearing CLs for efficient delivery of resveratrol to the posterior segment of the eye. To carry out the work, HEMA-based hydrogels were synthesized with 0, 190.5, and 381 mM MPC (Figure 1A). Wettability, state of water into the CL, mechanical properties, and MPC distribution into the CL through Raman spectroscopy were first characterized and compared to those of Proclear 1-day CLs. The loading and release profiles of resveratrol were evaluated before and after the sterilization of the CLs by steam heat. Searching for biorelevant release medium, the effect of adding proteins commonly present in the tear fluid on the release profiles was investigated and protein adsorption onto the CLs measured. The antibiofilm, anti-inflammatory, and antioxidant capabilities of most promising formulations were also studied. Then, after preliminary studies of biocompatibility, an *in vivo* test in New Zealand white rabbits was carried out to determine the safety and ocular tolerance of the developed CLs and the resveratrol accumulation in eye tissues. Finally, correlations between the release profiles of resveratrol *in vitro* and *in vivo* were investigated. To the best of our knowledge, this is the first time that MPC-based CLs are rationally designed considering the material properties, that resveratrol-loaded CLs are evaluated *in vivo*, and that *in vitro*–*in vivo* correlations (IVIVC) are attempted.

## Materials and Methods

### Materials

2-Hydroxyethyl methacrylate (HEMA), 3-(4,5-dimethylthiazol-2-yl)-2,5-diphenyltetrazolium bromide (MTT), and calcium chloride dihydrate (CaCl_2_·2H_2_O) were from Merck KGaA (Darmstadt, Germany). 2-Methacryloyloxyethyl phosphorylcholine (MPC), ethylene glycol dimethacrylate (EGDMA), dichlorodimethylsilane, 2,2′-azobis(2-methylpropionitrile) (AIBN), 2,2-diphenyl-1-picrylhydrazyl, and bovine serum albumin (BSA) were from Sigma-Aldrich (St. Louis, MO, USA). Lysozyme from egg white was from Fluka Analytical (Germany). Resveratrol was from ChemCruz, Santa Cruz Biotechnology Inc. (Dallas, TX, USA). Disodium hydrogen phosphate anhydrous (Na_2_HPO_4_) was from PanReac Química S.L.U. (Barcelona, Spain). Sodium chloride (NaCl) and hydrochloric acid 35% were from Labkem (Barcelona, Spain). Sodium bicarbonate (NaHCO_3_) was from Probus S.A. (Barcelona, Spain). Potassium chloride (KCl) was from Scharlab S.L. (Barcelona, Spain). Ethanol, absolute pure, was from PanReac AppliChem ITW Reagents (Barcelona, Spain). Methanol 99.9% for LC–MS grade was from Fisher Scientific (Loughborough, U.K.). Acetonitrile for HPLC LC–MS grade was from VWR Chemicals (Fontenary-sous-Bois, France). 2-Propanol and tryptic soy broth (TSB) were from Scharlau (Barcelona, Spain). Bacto tryptone was from Thermo Fisher Scientific (Detroit, MI, USA). Bacto yeast extract and Bacto agar were from Becton, Dickinson and Company (MD, USA). Fetal bovine serum, antibiotic solution (penicillin and streptomycin), lipopolysaccharides (LPS) from *Escherichia coli* 0111:B4, phorbol 12-myristate 13-acetate (PMA) and human tumor necrosis factor α (TNF-α) ELISA kit for serum, and plasma and cell culture supernatants were from Sigma-Aldrich (St. Louis, MO, USA). RayBio human interleukin-6 (IL-6) ELISA kit was from RayBiotech (Norcross, GA, USA). Ultrapure water (resistivity >18.2 MΩ·cm) was obtained by reverse osmosis (Milli-Q, Millipore Ibérica, Madrid, Spain). Tryptic soy broth (TSB-1), Luria–Bertani broth (LB), simulated lachrymal fluid (SLF) pH 7.4, and phosphate buffers were prepared as previously reported.^24^ Schirmer test strips were from Contactcare Ophthalmics and Diagnostics (Gujarat, India). Proclear 1-day CLs (Omafilcon A, CooperVision, CA, USA), diopter −3.00, water content 60%, Dk/t 28 were acquired from a local optical store.

### Preparation of HEMA Hydrogels and CLs

Various HEMA-based hydrogels with different amounts of MPC (Table 1) were prepared by adding 3 mL of HEMA to vials containing the corresponding amount of MPC, stirring (150 rpm; room temperature) for 1 h and then adding EGDMA (12.10 μL; cross-linker) and AIBN (32.85 mg; initiator). The mixture was kept under magnetic stirring (150 rpm, 30 min) for complete dissolution of AIBN. Then, the monomer solutions were injected through a 27 G needle into presilanized glass molds (12 cm × 14 cm) fixed with a 0.20 mm Teflon frame to carry out the polymerization at 50 °C for 12 h and 70 °C for other 24 h. The hydrogel sheets were demolded after polymerization, washed in 1 L of boiling distilled water for 15 min to remove unreacted monomers, and immediately cut into disks of 10 and 16 mm in diameter. The hydrogel disks were alternatively washed under magnetic stirring (130–200 rpm) in 500 mL of Milli-Q water and NaCl 0.9% solution, at least three times per day. Complete removal of unreacted monomers was monitored by measuring the absorbance of aliquots of the washing medium (UV–vis spectrophotometer Agilent 8453, Waldbronn, Germany). Finally, the disks were dried at 70 °C for 24 h.

**Table 1:** Composition of the Prepared Hydrogels and Solvent Uptake When the Swelling Equilibrium in Water, SLF, and Resveratrol Solution (100 μg/mL) in Ethanol/Water (10:90 v/v) Was Reached at Room Temperature^a^

hydrogel	HEMA (mL)	EGDMA (μL)	MPC (mg)	AIBN (mg)	water uptake (%)	SLF uptake (%)	ethanol/water uptake (%)
M0	3	12.10	0	32.85	58.5 (1.3)	55.8 (0.1)	73.5 (1.7)
M6	3	12.10	168.75	32.85	80.0 (0.8)	76.7 (0.6)	94.9 (1.0)
M12	3	12.10	337.50	32.85	103.8 (0.6)	103.1 (2.4)	119.1 (0.5)

a As a reference, the solvent uptake of Proclear 1-day CL was 139.9% (2.3), 150.8% (2.9), and 174.5% (6.5) for the same three media, respectively. M12 prepared as CLs had the same solvent uptake values as in form of disks. Listed are mean values and, in parentheses, standard deviation (*n* = 2).

**Figure 2:**
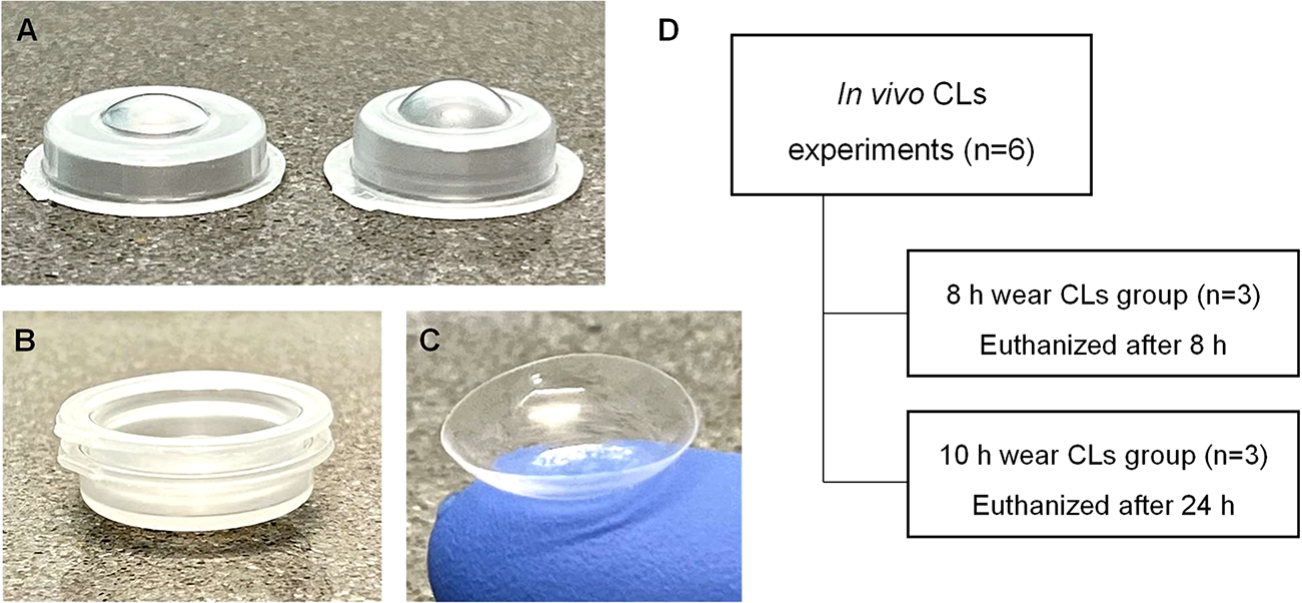
(A, B) Curved polypropylene molds used to prepare the contact lenses, (C) image of a contact lens as removed from the molds, and (D) distribution of the animals for the *in vivo* experiments.

CLs were synthesized by adding 60 μL of the monomers solution with the highest amount of MPC (M12) into curved polypropylene molds typically used for preparing daily disposable CLs (*n* = 20) (Figure 2A–C). The polymerization and washing of all CLs were carried out as described above for the hydrogel disks. The dimensions of the CLs obtained in the hydrated state in phosphate buffer pH 7.4 (107.4 ± 1.6% water content) were 14.1 ± 0.1 mm diameter, 8.8 ± 0.1 mm curvature, and 0.1 ± 0.0 mm thickness. Finally, the CLs were washed in 1 L of Milli-Q water to remove the phosphate buffer pH 7.4 and dried at 40 °C for 2 h and at 70 °C for another 2 h to be used in further experiments.

### Hydrogels Characterization

Raman spectra of dried disks were recorded in a Alpha300 R+ Raman imaging microscope fitted with a 532 nm laser (WITec GmbH, Ulm, Germany).^34^ Additionally, distribution of MPC along the thickness of disks and CLs was recorded by monitoring the peak at 717 cm^–1^ for a 25 μm line along a depth of 85 μm (120 lines; 75 spectra per line; 9000 spectra in total).

The solvent uptake was quantified, in duplicate, as the increase in weight of dried hydrogel disks (10 mm diameter) or CLs when placed in vials containing 5 mL of water, SLF, or resveratrol loading solution (100 μg/mL in ethanol/water 10:90 v/v) at room temperature and protected from light. The disks were weighed at preset time points (1, 2, 3, 4, 5, 6, 7, 8, 24, 48, and 120 h) after carefully removing the excess liquid on their surfaces with absorbent paper. The solvent uptake was calculated through the following equation where *W*_0_ and *W*_*t*_ represent the initial weight and at time *t* weight of the hydrogel.
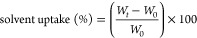
1After the swelling, the transmittance (%) of all hydrogels was measured from 200 to 700 nm with 1 nm intervals (Agilent Cary 60 UV–vis, Waldbronn, Germany).

Differential scanning calorimetry (DSC Q100 TA Instruments, New Castle, DE, USA) was used to determine the glass transition temperature, *T*_g_, of the dried disks (3–4 mg samples in nonsealed aluminum pans) by heating from 40 to 140 °C, cooling to −20 °C, and finally heating again up to 300 °C, at 10 °C/min. The proportion of freezing (unbound) water in water-swollen hydrogels was quantified using hydrogel pieces sealed in aluminum pans that were cooled to −60 °C and then heated to 60 °C, at 5 °C/min.^35,36^ The total content in water of the hydrogels was assumed to be the sum of the three water states (nonfreezing water, *W*_nf_, freezing bound water, *W*_fb_, and free water, *W*_f_) as follows:
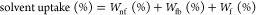
2The sum of *W*_fb_ (%) and *W*_f_ (%), i.e., the freezable water, was estimated from the enthalpy of ice–water melting in the sample. The melting enthalpy of pure water was also determined and used as reference. All DSC experiments were carried out at least in duplicate under a nitrogen flow rate of 50 mL/min after calibration of the equipment with indium (melting point 156.61 °C, enthalpy of fusion 28.71 J/g).

Mechanical properties of water-swollen M0, M6, and M12 hydrogels (strips 16 mm × 9 mm) were recorded, in triplicate, in a TA.XT Plus texture analyzer (Stable Micro Systems, Ltd., Surrey, U.K.) fitted with a 5 kg load cell. The strips were fixed to the lower and the upper clamps (7 mm gap) and subjected to uniaxial stress at 0.1 mm/s. Young’s modulus was calculated from the slope of the linear portion of the engineered stress versus engineered strain curves.

### Loading of Resveratrol in Nonsterilized and Sterilized Hydrogels and CLs

The loading of resveratrol was carried out for both nonsterilized and sterilized (steam heat 121 °C, 20 min; Raypa steam sterilizer, Terrassa, Spain) hydrogel disks (10 mm diameter, average weight 16 mg) and CLs (17–24 mg weight) in 15 mL Falcon tubes containing 7 mL of a resveratrol loading solution (100 μg/mL in ethanol/water 10:90 v/v). The Falcon tubes were kept protected from light to avoid resveratrol degradation, at 36 °C and 180 rpm for 24–72 h. The absorbance of the loading medium was measured every 2 h for the first 8 h and then every 24 h at 305 nm (Figure 1B; UV–vis spectrophotometer Agilent 8534, Waldbronn, Germany) after leveling the aliquots (250 μL for the first 8 h and 500 μL after) to 5 mL with ethanol/water 10:90 v/v. To evaluate the effects of sterilization, dried disks and CLs were sterilized in 15 mL Falcon tubes (steam heat 121 °C, 20 min). In parallel, the resveratrol loading solution was sterilized by filtration (Filter-Lab polyethersulfone, PES, syringe filter 0.22 μm; Barcelona, Spain) and collected in the tubes under sterile conditions in a biological safety cabinet. Resveratrol loading by sterile disks was monitored both with and without intermediate measurements for 96 h to check a possible effect on the loading due to the amount of resveratrol lost in the monitoring. All experiments were carried out in quadruplicate. The loading of sterile CLs was carried out in various batches, at least in triplicate, as explained above at 36 °C and 180 rpm without intermediate measurements. The amount of resveratrol loaded was assessed from the difference between the initial and final amounts of resveratrol in the loading solution using a validated calibration curve of resveratrol in the same medium and considering the amount of resveratrol lost in the monitoring. The network/water partition coefficient (*K*_N/W_) of resveratrol was calculated as previously described.^37^

### *In Vitro* Release of Resveratrol from Loaded Hydrogels and CLs

The release from both sterilized and nonsterilized formulations was evaluated in the same way. After the loading, the disks and CLs were rinsed with NaCl 0.9% and placed into 15 mL Falcon tubes with 6 mL of NaCl 0.9%. The release was carried out protected from light at 36 °C and 180 rpm for at least 24 h in an incubating shaker (Incubator 1000, Heidolph, Germany). The absorbance of the release medium was measured at 305 nm (UV–vis spectrophotometer Agilent 8534, Waldbronn, Germany) by taking aliquots of 3 mL in the first 2 h that were immediately returned to the Falcon tubes after the measurement. After that, aliquots of 1 mL were taken at preestablished times, which were replaced with the same volume of NaCl 0.9% fresh solution. After measurement of the absorbance at 8 h, 6 mL more of NaCl 0.9% fresh solution was added increasing the release medium in all tubes to 12 mL to avoid the saturation of the medium and false plateaus. The amount of resveratrol released was quantified using a validated calibration curve of resveratrol in NaCl 0.9% and considering the amounts removed and the corresponding dilution of the samples. Higuchi equation was fitted to individual release profiles (10–60% released) to estimate the release rate constant, *K*_H_, as follows,^35^
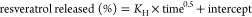
3

Release experiments in the presence of lysozyme and BSA were also carried out by immersing the resveratrol-loaded sterilized CLs in 6 mL of NaCl 0.9% with or without 2.68 mg/mL of BSA and lysozyme.^38^ The release experiment was carried out following the protocol described above but removing aliquots (200 μL) of the release medium at each time and replacing with the same volume of fresh medium (NaCl 0.9% or NaCl 0.9% plus lysozyme or BSA). Protein denaturation was carried out by heating the aliquots at 98 °C for 2 min, cooling them in an ice bath for 10 min, and centrifuging at 13 000 rpm for 10 min at 25 °C. Finally, the supernatants were collected and stored at −20 °C until HPLC analysis. Aliquots without protein were processed in the same way. The amount of resveratrol released was quantified previous dilution of the samples in ethanol/water 50:50 v/v, using a JASCO (Tokyo, Japan) HPLC (AS-4140 autosampler, PU-4180 pump, LC-NetII/ADC interface box, CO-4060 column oven, MD-4010 photodiode array detector), fitted with a C18 column (Waters Symmetry C18, 5 μm, 4.6 mm × 250 mm) and operated with ChromNAV software (JASCO, Tokyo, Japan). The analysis was carried out by isocratic elution using a mobile phase of methanol/water 50:50 v/v at a flow rate of 1 mL/min, 35 °C and with 8 min of run time. The injection volume was 50 μL, and the UV detector was set at 305 nm. Retention time was 4.6 min. Validation of the method was performed using a calibration curve of resveratrol in ethanol/water 50:50 v/v in the 0.05–6 μg/mL range. All release experiments were performed at least in triplicate.

In parallel, the amounts of lysozyme and BSA sorbed onto previously hydrated (7 mL of ethanol/water 10:90 v/v, 36 °C, 180 rpm for 48 h) M0, M6, and M12 disks and Proclear 1-day CLs were recorded during 24 h after immersion in a lysozyme (0.5 mg/mL; 6 mL) or BSA (2.68 mg/mL; 6 mL) solution in NaCl 0.9% by monitoring the absorbance of the medium at 280 nm (UV–vis spectrophotometer Agilent 8534, Waldbronn, Germany). After measurement each aliquot was returned to the original vial (*n* = 3). A validated calibration curve in NaCl 0.9% was used for each protein in the 0.134–2.68 mg/mL range for BSA and 0.1–0.5 mg/mL range for lysozyme.

### HET-CAM Test

The hen’s egg test on the chorioallantoic membrane (HET-CAM) was performed to assess the potential ocular irritation capacity of all developed resveratrol-loaded hydrogels as previously described.^37^ The hydrogel disks (M0, M6, and M12) were loaded as described above and placed on the eighth day on the CAM of the fertilized and incubated eggs after removing the inner membrane. The eggs were monitored for 5 min regarding hemorrhage, vascular lysis, and coagulation of the CAM vessels to calculate the irritation score (IS) as previously reported.^39^ 0.9% NaCl and 0.1 N NaOH solutions (300 μL) were used as negative and positive controls, respectively.

### Antibiofilm Properties

The capacity of blank and resveratrol-loaded M0 and M12 hydrogels to inhibit biofilm formation by two common bacteria causal agents of ocular infections, i.e., *Pseudomonas aeruginosa* (PAO1, Lausanne subline, donated by M. Cámara, University of Nottingham) and *Staphylococcus aureus* (ATCC25923, Manassas, VA, USA), was evaluated in a novel agar–CL interface biofilm growth method that aimed to resemble bacterial growth conditions in the CL–eye interface (Figure S1 in Supporting Information). All experiments were performed in triplicate in a biological safety cabinet under sterile conditions. *P. aeruginosa* and *S. aureus* were regularly grown (37 °C, 24 h) in LB and TSB-1, respectively. The preinocula and inocula preparation was carried out following a slightly modified previous protocol^24,40^ by inoculating a 24 h plate colony of the corresponding bacteria in 10 mL of culture medium at 37 °C and 100 rpm for 12 h (*P. aeruginosa*) or 24 h (*S. aureus*). Then, aliquots (1 mL) of both preinocula were centrifuged in duplicate at 13 000 rpm for 3 min and resuspended in 1 mL of fresh medium before adjusting their optical density at 600 nm (UV–vis spectrophotometer Thermo Scientific Helios Omega) to 0.05 (*S. aureus*) or 0.01 (*P. aeruginosa*) by dilution into 50 mL Falcon tubes containing the corresponding culture medium.

#### Plates Preparation and Incubation

Wells of 24-wells cell-culture plates were filled with 2 mL of sterilized tryptic soy agar supplemented with 1% NaCl (TSA-1) or LB agar (1.5% agar final concentration in both cases). The plates were left to dry under sterile conditions for approximately 1 h and stored at room temperature until their use. The antibiofilm capacity of both blank and resveratrol-loaded hydrogels was studied after 12 h of growth for both bacteria. The sterile hydrogels (steam heat 121 °C, 20 min) were soaked in a previously filtered (Biofil syringe filter, 0.22 μm PES membrane; Barcelona, Spain) resveratrol (100 μg/mL) solution in ethanol/water 10:90 v/v (7 mL, 36 °C, 180 rpm for 4 days). Hydrogels without resveratrol were treated in the same way and soaked in 7 mL of ethanol/water 10:90 v/v. The amount of resveratrol loaded was estimated from the absorbance measured at 305 nm (as above). Then, 20 μL of the corresponding bacterial inoculum was placed in the center of each well and, just above it, the hydrogel disks were carefully placed as shown in Figure S1 (Supporting Information) in order to facilitate the growth of the bacteria on the interface hydrogel/agar and to resemble the releasing conditions in a limited aqueous medium as occurs in the CL–eye interface. The plates were incubated at 37 °C for 12 h protected from light for both bacteria. Biofilm formation on the hydrogel disks surface was evaluated (in triplicate) using the MTT assay and the determination of colony-forming units (CFUs) after washing the disks with 2 mL of PBS in a new 24-well plate, as described in the Supporting Information.

### Anti-Inflammatory Activity

The anti-inflammatory activity of nonloaded and resveratrol-loaded M0 and M12 sterilized hydrogels was evaluated using THP-1 human monocytes (ATCC TIB-202; ATCC, Manassas, VA, USA). The hydrogels were sterilized and loaded with resveratrol as described below for *in vivo* experiments. Nonloaded hydrogels were treated in the same way and soaked in an ethanol/water 10:90 v/v solution without resveratrol. RPMI 1640 medium (Gibco Thermo Fisher Scientific; Newington, NH, USA) supplemented with 10% fetal bovine serum and 1% penicillin–streptomycin was used to culture the monocytes at 37 °C, 5% CO_2_, and 90% relative humidity. Then, differentiation into macrophages was carried out by adding PMA (400 nM) for 72 h at 37 °C. After differentiation, the PMA solution was removed, and the macrophage cell monolayers were washed with DPBS and trypsinized to be immediately later seeded (50 000 cells/well) into a 24-well plate for at least 6 h. Given the adherent capacity of macrophages, a specific cell scraper was used to remove all adherent cells. After this, one piece of nonloaded and resveratrol-loaded M0 and M12 hydrogel disks was added to each well. Resveratrol 25 and 50 μM solutions were also tested in parallel. These solutions were prepared 10 times more concentrated in ethanol/water 10:90 v/v (250 and 500 μM) given the low solubility of resveratrol in water and the dilution factor suffered after addition in the culture medium. Therefore, to avoid false anti-inflammatory outcomes due to the presence of ethanol during the test, the effect of ethanol/water 10:90 v/v mixture was also studied (final percentage of ethanol was 1% after dilution in the culture medium). After overnight incubation, cells were stimulated with LPS (100 ng/mL) and incubated for 24 h at 37 °C, 5% CO_2_, and 90% relative humidity. No-stimulated (without LPS) and stimulated (with LPS) cells acted as negative and positive controls, respectively. Finally, after 24 h incubation, cell culture supernatants were collected, and the secretion of TNF-α and IL-6 was analyzed by specific ELISA kits following manufacturer protocols.

### *In Vivo* Experiments: Resveratrol Release, and Accumulation in the Ocular Tissues

#### Animal Groups

All *in vivo* experiments fulfilled 3R’s principles and were carried out following the Association for Research in Vision and Ophthalmology (ARVO) Statement for the Use of Animals in Ophthalmic and Vision Research and the European Directive 2010/63/EU, with the corresponding permission of the Ethics Committee for Animal Experimentation of the University Complutense of Madrid [registration number: O00023280e2100023620]. Male New Zealand white rabbits (age approximately 3 months and 4.11 ± 0.53 kg weight) were stabled in individual cages with total access to food and water at 18 °C and 50% relative humidity inside a light-controlled room with 12 h light–dark cycles. Rabbits with low weight, illness, or corneal disruptions were discarded. The rabbits (*n* = 6) were divided into two groups: one group of rabbits (*n* = 3) wore the CLs for 8 h and were euthanized after this period, and the other group (*n* = 3) wore the CLs for 10 h and were euthanized after 24 h (Figure 2D). To minimize the effects of subjective bias, the experiments were carried out in 3 days and the rabbits where randomly assigned to each assay day. All experiments started at 7:30–8:30 am. No animals or data were discarded. No adverse events were detected. These *in vivo* experiments were carried out under the same conditions as those reported for resveratrol-loaded Pluronic F127 micelle eye-drops prepared with a resveratrol concentration of 4 mg/mL and a Pluronic F127 concentration of 10 mM. For the eye drops, a single drop (50 μL; 200 μg of resveratrol) was gently instilled in the lower conjunctival sac of the right eye using a micropipette.^41^ The amount of resveratrol loaded by each CL M12 tested *in vivo* was also approximately 200 μg.

#### *In Vivo* Release Tests

Dried CLs were sterilized and loaded for 72 h (8 h wear CLs group) or 48 h (10 h wear CLs group) as described above. Then, the CLs were removed from the loading solution, rinsed with sterile saline solution (Avizor sterile saline unidose 5 mL), and placed on the right eye cornea below the nictitating membrane without local anesthesia. The rabbits were maintained in rabbit restrainers with continuous monitoring to not remove the CLs placed on the ocular surface. Moreover, the eyes of the rabbits were closed every few minutes to ensure the hydration of the CLs.

The ocular surface of the rabbits was carefully observed with a VX75 slit lamp (Luneau Technology, Chartres, France) before and after the treatment. Samples of tear fluid were collected before and after CL wearing (*t* = 5 min, 15 min, 30 min, and every hour for 8–10 h) using Schirmer test strips which were placed in the tarsal conjunctiva of the inferior lid for 10 s with closed eyes to avoid the reflex secretion associated with blinking.^42^ The volume collected was calculated as a function of the millimeters of wetted strip. Left eyes of all rabbits acted as controls (without treatment).

#### Quantification of Resveratrol in the Tear Fluid

The Schirmer test strips were cut into small pieces and placed protected from light into 1.5 mL Eppendorf tubes to which 200 μL of ethanol/water 50:50 v/v solution was added. The tubes were vortexed for 1 min and left in the fridge at 4 °C for approximately 15 h. Then, the samples were vortexed again for 2 min and the strips removed from the tubes. Protein denaturation and HPLC analysis were performed as described above after freezing the samples at −80 °C. The extraction and protein denaturation method were shown to reproducibly recover more than 98% resveratrol present in the samples.

#### *In Vitro*–*In Vivo* Correlations (IVIVCs)

IVIVCs were investigated using Levy plot analysis, representing the percentage of resveratrol released *in vitro* at a certain time (*X*-axis) vs the percentage of resveratrol released in the lachrymal fluid at the same time (*Y*-axis) calculated as cumulative area under the tear fluid concentration–time curve normalized as percent of the total area using the following equation:^43^
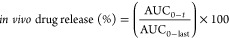
4

#### Quantification of Resveratrol in the Ocular Tissues

After the experiments, all rabbits were euthanized by intravenous injection of 0.75 mL/kg of propofol and 0.5 mL/kg of pentobarbital sodium (Exagon 400 mg/mL). Then, the aqueous humor of both eyes was directly extracted from the anterior chamber with an insulin needle in 1.5 mL Eppendorf tubes. The eyes were enucleated and immediately stored in 50 mL Falcon tubes at −80 °C until being dissected. The dissection was carried out separating cornea, crystalline lens, vitreous humor, retina, and sclera in 1.5 mL Eppendorf tubes. Ethanol/water 50:50 v/v was added to cornea (500 μL), crystalline lens (500 μL), sclera (800 μL), and retina (200 μL). All Eppendorf tubes were kept in the fridge protected from light at 4 °C for 12 h, and then the protein denaturation process was carried out as described above. The supernatants were stored at −80 °C until UPLC analysis. Before UPLC analysis all samples were diluted 1.5 times with acetonitrile and mixed using an automated liquid handling system, Caliper Zephyr, 3 cycles of 50 at 78 μL/s. The plate was then centrifuged at 3700 rpm at 4 °C for 30 min. The quantification of resveratrol in the different tissues was carried out using a Waters Acquity UPLC H-class coupled with a Xevo TQD MS system fitted with an HypersilGOLD C18 column (1.9 μm, 2.1 mm × 50 mm, Thermo-Fischer) with a column temperature of 35 °C. The analysis was done by gradient elution using water + 0.1% formic acid as solvent A and using acetonitrile + 0.1% formic acid as solvent B, as follows: 0–0.1 min 5% B, 0.1–1.0 min 5–100% B, 1.0–2.0 min 100% B, 2.0–2.1 min 100–95% B, and 2.1–2.5 min 5% B and at a flow rate of 0.6 mL/min. Electrospray ionization (ESI) was run in positive mode with a source temperature of 150 °C and a desolvation temperature of 500 °C. Capillary voltage was set to 3 kV, and the cone voltage was set to 45 V. Desolvation gas flow was 900 L/h, and cone gas flow was set to 50 L/h. The compound of interest was monitored using multiple reaction monitoring (MRM)/ES+ mode Precursor, and product ion transition for analyte resveratrol 229.045 > 106.993 was monitored. The injection volume was 4 μL and the retention time was 1.18 min. Typical UPLC chromatograms are depicted in Figure S2 (Supporting Information).

### Quantification of the Antioxidant Activity of Resveratrol Remaining in the CLs after *in Vivo* Wearing

After the *in vivo* experiment the CLs were stored in closed vials at room temperature and protected from light for a few days to be later immersed in 3 mL of ethanol/water 50:50 v/v solution to extract the remnant amount of resveratrol inside the lenses at 36 °C and 180 rpm for 4 h (Incubator 1000, Heidolph, Germany). To carry out the test, a 0.026 mM DPPH solution (50 mL) in ethanol was freshly prepared and stored protected from light. Then, an aliquot of the extraction medium (500 μL) was mixed with the same volume of DPPH solution and vortexed for 5 s. The absorbance was measured at 517 nm (UV–vis spectrophotometer Agilent 8534, Germany) after 30 min of incubation in darkness. The antioxidant activity of the ethanol/water 50:50 v/v solution without resveratrol was also measured and served as control. The test was carried out in triplicate for 8 h wear CLs, 10 h wear CLs, and controls. The DPPH scavenging capacity was expressed as μg/mL of DPPH in the reaction medium calculated from a previously developed and validated calibration curve of DPPH in ethanol (2.56–20.5 μg/mL) and as DPPH scavenging effect in percentage as follows,^24^
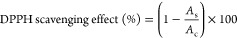
5In this equation, *A*_s_ and *A*_c_ represent the absorbance at 517 nm of the extraction medium and the control, respectively.

### Statistical Analysis

The statistical analysis was performed using the Statgraphics Centurion 18, version 18.1.13 software (StatPoint Technologies Inc., Warrenton VA, USA). Differences between formulations on resveratrol release rate *in vitro*, antimicrobial performance, anti-inflammatory activity, and resveratrol levels in tear fluid and eye tissues were analyzed using ANOVA for independent samples and multiple range tests. A statistical significance of 95% (*p* < 0.05) was established in all the statistical tests, while the results are expressed as the mean ± standard deviation.

## Results and Discussion

### Preparation and Structural Characterization of Hydrogels and CLs

Three different HEMA-based hydrogels were designed varying the content in MPC (Table 1) to elucidate the effect of this variable on hydrogel wettability and capability to uptake resveratrol, with the aim of improving the antibiofilm and antioxidant features of HEMA-based CLs. All hydrogel disks were polymerized into molds with the lowest thickness as possible (0.2 mm) to try resembling the CLs thickness (0.1 mm). The CLs were synthesized with the highest amount of MPC, i.e., 381 mM (designed as CL M12), which was 3.8 times greater than the proportion tested before^24^ and the proportion disclosed for the commercially available Proclear CLs.^23^ MPC has been claimed to improve the comfort of the CLs due to its capacity to enhance the wettability and biocompatibility of the material, to protect corneal cells from damage, and to decrease protein adsorption and microorganism adherence.^22,44,45^ In the present work, unlike most of previously published reports, the incorporation of MPC was carried out by adding it as a comonomer during the synthesis and not as a surface component of post-synthesis-modified materials.^18,46,47^ The chosen MPC proportions easily dissolved in the HEMA solution under magnetic stirring at 150 rpm for 1 h. MPC proportions above 400 mM required a long time to be dissolved and thus were discarded. Hydrogel codes M0, M6, and M12 in Table 1 corresponded to 0, 190.5, and 381 mM MPC, namely, 0, 6, and 12% MPC.

**Figure 3:**
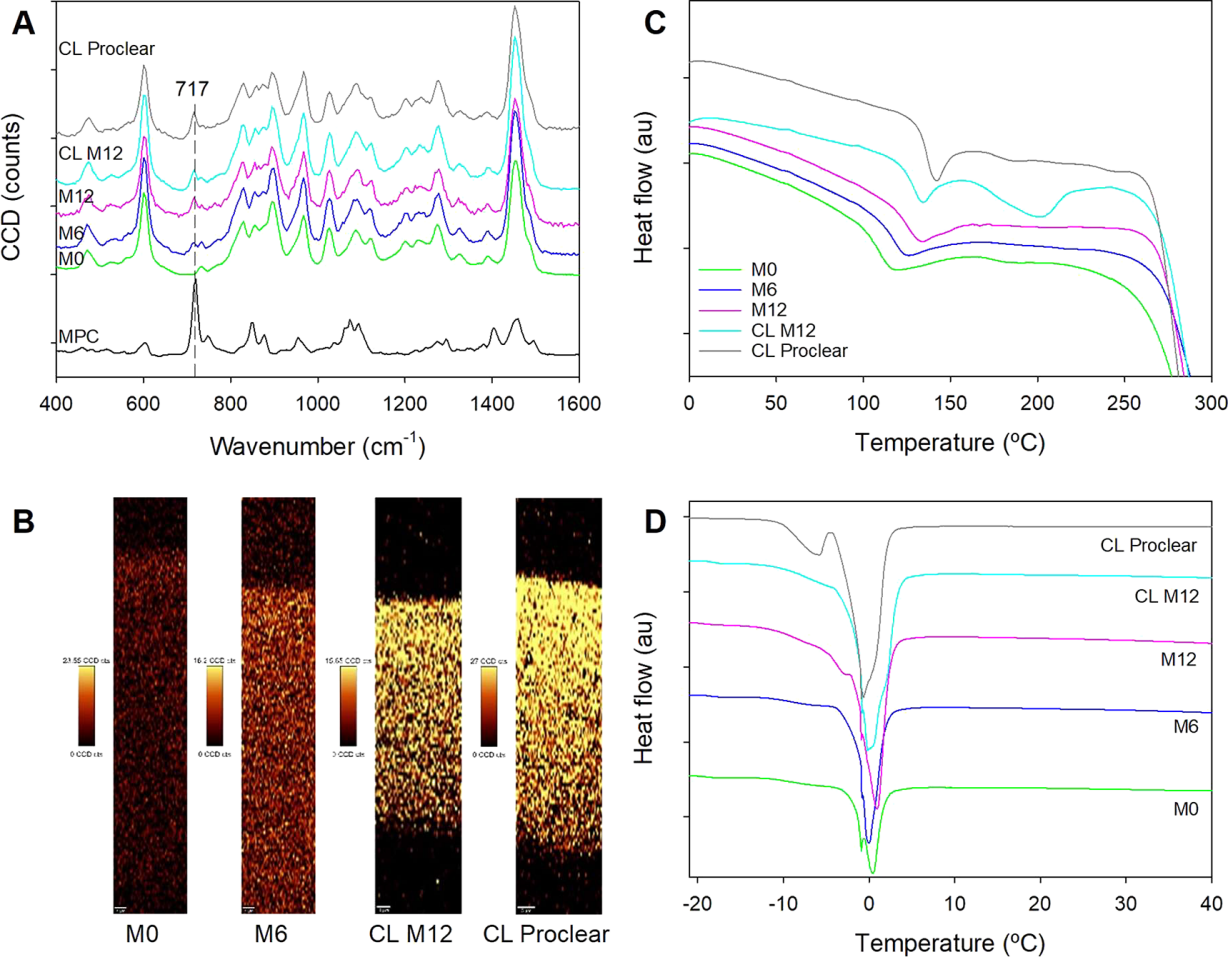
(A) Raman spectra of dried hydrogels and CLs, including the spectrum of MPC monomer as a reference; (B) Raman microscope images of dried disks and CLs (along the depth) recorded with a filter at 717 cm^–1^, showing the increase in MPC signal in a red to yellow scale; (C) DSC scans recorded during the second heating of dried disks and CLs (after heating to 120 °C and cooling to −20 °C at a rate of 5 °C/min); (D) DSC scans recorded for hydrated disks and CLs after being cooled to −60 °C. Hydrogel codes are as in Table 1.

Raman analysis confirmed the presence of MPC and its homogeneous distribution along the thickness of the hydrogel disks and CLs (Figure 3A,B). MPC showed a distinctive peak at 717 cm^–1^ due to the CN^+^ band^48^ whose relative intensity increased from M0 to M12. This peak was used to visualize in the Raman microscope the distribution of the monomer. Although the technique was semiquantitative, a clear increase in the intensity of the signal was observed as the content of MPC in the hydrogels increased, and the distribution of MPC in the bulk of the disks and CLs was evidenced (Figure 3B).

The *T*_g_ of dried M0 disks was 107 °C (Figure 3 C, Table 2) in good agreement with previous reports on HEMA-based networks.^35^ An increase in MPC content shifted the *T*_g_ values toward higher temperature, which indicated an increase in the stiffness of the networks. It should be noted that all samples were subjected to heating–cooling cycles under the same conditions, and the *T*_g_ analysis was carried out during the second heating. CL M12 and Proclear CLs were the ones with the higher *T*_g_ values (thermodynamic component) and showed a clear enthalpy relaxation (kinetic process). The endothermic relaxation suggested that below the *T*_g_ and for the experimental cooling/heating rate of 5 °C/min there was a relaxation of the cooled network, and the polymeric chains still had some mobility. Such an increased chain mobility below the *T*_g_ observed for the CLs (M12 and Proclear) may be related to either the smaller thickness (0.1 mm compared to 0.2 mm of disks) or a lower cross-linking degree.^49^

**Table 2:** Glass Transition Temperature (*T*_g_), Ice Melting Temperature (*T*_m_), and Enthalpies Referred to the Total Weight of the Sample (Δ*H*_exp_) and to the Content in Water (Δ*H*_m_), Freezable Water, Young’s Modulus of the Water-Swollen Hydrogels, and the Network/Water Partition Coefficient of Resveratrol (*K*_N/W_)^a^

hydrogel	*T*_g_ (°C)	Δ*H*_exp_(J/g)	*T*_m_ (°C)	Δ*H*_m_(J/g)	freezable water (%)	Young’s modulus (MPa)	*K*_N/W_
M0	107.0	10.09	0.05	27.34	7.89	0.69 (0.02)	114.5 (0.1)
M6	112.4	17.47	0.18	39.35	11.36	0.60 (0.01)	124.8 (0.4)
M12	124.1	37.40	0.33	73.77	21.30	0.51 (0.01)	122.3 (1.3)
CL M12	129.0	40.83	–0.59	80.82	23.33		111.8 (1.8)
CL Proclear	136.7	43.48	–0.39	74.96	21.64		124.5 (1.1)

a As a reference, the enthalpy of free water was determined to be 346.4 J/g. Listed are mean values (*n* = 3) and, in parentheses, the standard deviations. The standard deviations of DSC-related parameters were below 10%.

All hydrogels were characterized regarding solvent uptake and transmittance to fulfill the requirements demanded by commercially available CLs. The solvent uptake (Figure S3, Supporting Information) was evaluated in water, SLF, and resveratrol loading solution (100 μg/mL in ethanol/water 10:90 v/v) and significantly increased as the content of MPC in the hydrogels increased (Table 1). The solvent uptake occurred very rapidly, and no differences were registered between water and SLF for a given composition. Differently, the presence of ethanol in the resveratrol loading solution notably increased the swelling as previously observed.^24^ M12 hydrogel disks showed the highest water uptake value (103.8 ± 0.6%).

The values registered for CL M12 were slightly lower than the solvent uptake of Proclear CL, which was 139.9% (2.3), 150.8% (2.9), and 174.5% (6.5) in water, SLF, and ethanol/water (10:90 v/v), respectively, which may be because the prepared CL M12 had a higher content in the cross-linking agent EGDMA. The proportion of EGDMA in Proclear CLs has not been disclosed, but their higher swelling even containing less MPC than CL M12 and the intense enthalpy relaxation recorded in the DSC scans corroborated the hypothesis of their lower cross-linking density. Regarding light transmission, all hydrogels were optically transparent in the 500–700 nm range as required for CLs (Figure S4, Supporting Information). The transmission in the UV region was blocked after resveratrol loading, which means that the antioxidant acted as an efficient UV filter, in good agreement with its UV–vis spectrum shown in Figure 1B.

Since the interaction of CL networks with water may determine the interface properties with the host (tear fluid components and cornea of the wearer) and the microorganisms,^50^ the state of water in the swollen MPC hydrogels was further investigated applying DSC (Figure 3D). The enthalpy of free water was determined to be 346.4 J/g in good agreement with previous reports.^35^ The percentage of free water in the hydrogels (Table 2) showed a positive correlation with the content in MPC; hydrogel M12, CL M12, and Proclear CLs had more than 20% free water, and nonsignificant differences were observed among them. These findings confirmed that MPC notably increased the proportion of free (movable) water in the hydrogels.

The presence of MPC caused a clear softening of the swollen hydrogels, which was shown as a significant decrease in Young’s modulus (Table 2). Therefore, copolymerization of HEMA with MPC rendered hydrogels in the range of the contact lenses with the lowest Young’s modulus and thus the most comfortable ones.^50^

### Resveratrol Loading Tests

**Figure 4:**
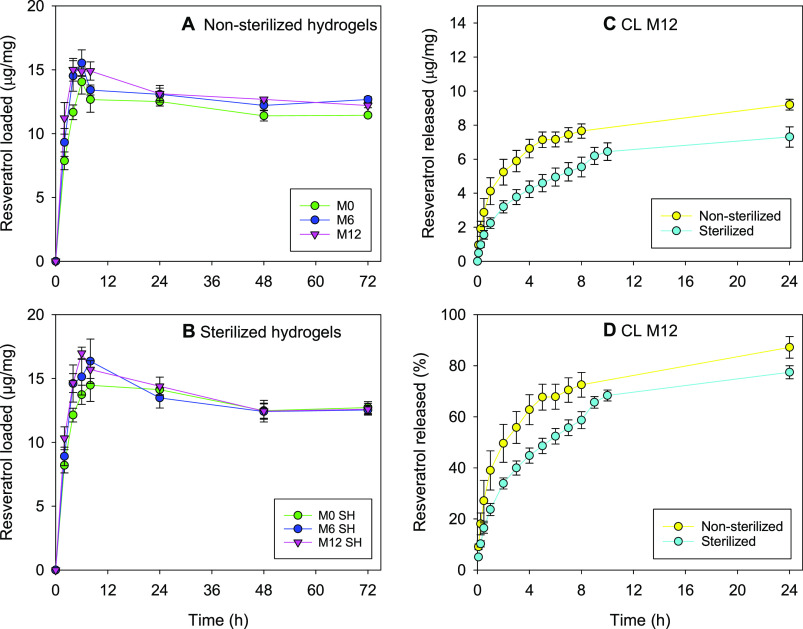
(A, B) Resveratrol loading profiles recorded for (A) nonsterilized and (B) sterilized hydrogel disks and (C, D) resveratrol release profiles in NaCl 0.9% protected from light at 36 °C and 180 rpm from nonsterilized and sterilized CL M12. Shown are mean values and standard deviation (*n* = 3). Codes are as in Table 1.

Since CLs must be sterile but resveratrol did not withstand the typical steam heat sterilization process,^51^ a two-step sterilization protocol was implemented with the hydrogels. First, the hydrogels were steam-heat sterilized in empty vials, and then the vials were aseptic filled with the resveratrol solution (100 μg/mL in ethanol/water 10:90 v/v). The capability of all hydrogels to load resveratrol was evaluated for both nonsterilized and steam heat sterilized disks. All formulations followed a similar loading pattern, and after 48 h of soaking, the amount of resveratrol loaded was similar for all hydrogel types (Figure 4A,B; raw data available in Table S1). Relevantly, the increase in MPC proportion and thus in water uptake did not compromise the loading capacity expressed as the total amount of resveratrol loaded referred to dried mass of hydrogel. Regarding the encapsulation efficiency, estimated as the amount of resveratrol loaded with respect to the initial amount in the loading solution, the hydrogels ranked as follows: M0 (28.8 ± 0.4%) ≅ M6 (28.3 ± 0.5%) > M12 (23.6 ± 0.3%). The minor differences found could be attributed to small differences in dried mass of the hydrogels; namely, an increase in MPC determined that the same disk size had lower dried mass.

Similarly, no differences in total amount of resveratrol loaded were registered between nonsterilized (10.56 ± 0.20 μg/mg) (Figure S5 in Supporting Information) and sterilized CLs for 24 h (10.73 ± 0.96 μg/mg) or 48 h (11.45 ± 0.78 μg/mg) loading. In addition, intermediate sampling with the consequent loss of minor amounts of resveratrol from the loading medium did not significantly affect the total amount loaded. The similar loading of resveratrol by all hydrogels disregarding the content in MPC and, thus, in water may be explained by a nonspecific hydrophobic binding of resveratrol to the pHEMA network. The amounts of resveratrol that could be loaded in the aqueous phase of hydrogels clearly increased as the content in MPC increased, ranking in the order M0 (0.058 ± 0.001 μg/mg) < M6 (0.080 ± 0.001 μg/mg) < M12 (0.104 ± 0.001 μg/mg) ≅ CL M12 (0.101 ± 0.002 μg/mg) < CL Proclear (0.139 ± 0.003 μg/mg). However, such an increase had negligible impact on the total loading, which was 2 orders of magnitude larger. Thus, the affinity of resveratrol for the network was markedly greater than for the aqueous phase, which led to *K*_N/W_ values in the 111–125 range (Table 2). Relevantly, MPC had no detrimental effects on the binding of resveratrol. Compared to other antioxidant agents, such as ferulic acid which had *K*_N/W_ values of 17.9,^25^ resveratrol was more prone to bind to the pHEMA network, which favored the loading and could provide a more sustained release.

### *In Vitro* Release of Resveratrol

The release of resveratrol was tested using both sterilized and nonsterilized hydrogels and with/without the presence of proteins in the release medium for CL M12. The volume of the release medium (initially 6 mL) was incremented to 12 mL after 8 h to avoid saturation and artifact plateaus considering the low solubility of resveratrol in NaCl 0.9% (27.4 ± 1.4 μg/mL). All hydrogel disks (Figure S6 in Supporting Information; raw data available in Table S2) as well as the CLs (Figure 4C,D) showed sustained release patterns *in vitro* that fitted to the Higuchi kinetics (*r*^2^ > 0.99). In the case of the disks, the release rates estimated from the first 8 h release time were 2.08 (sd 0.18) % min^–0.5^ for M0 nonsterilized, 2.22 (sd 0.09) % min^–0.5^ for M0 sterilized, 2.30 (sd 0.96) % min^–0.5^ for M6 nonsterilized, 2.26 (sd 0.32) % min^–0.5^ for M6 sterilized, 2.92 (sd 0.31) % min^–0.5^ for M12 nonsterilized, and 2.84 (sd 0.22) % min^–0.5^ for M12 sterilized. No differences were observed between sterilized and nonsterilized hydrogels. Differently, a significant increase in resveratrol release rate was observed for M12 hydrogels (either sterilized or nonsterilized) compared to M0 and M6 (ANOVA, *p* < 0.005). This finding is in good agreement with the greater swelling and higher content in free water of M12 hydrogels, which facilitate the diffusion of resveratrol through the network. Despite the high hydrophilicity of the hydrogels prepared with the higher content in MPC (i.e., M12 hydrogels), the affinity of resveratrol for the HEMA network still efficiently regulated the release process.

In the case of CL M12, although the loading was similar, the total amount of resveratrol released at 24 h was higher for nonsterilized (9.20 ± 0.31 μg/mg) than for sterilized CLs (7.31 ± 0.59 μg/mg) (Figure 4C,D); the release rates estimated from the first 6 h release time were 3.80 (sd 0.32) % min^–0.5^ for CL M12 nonsterilized and 2.84 (sd 0.15) % min^–0.5^ for CL M12 sterilized. Proclear CLs showed resveratrol release profiles^25^ superimposable to those of nonautoclaved CL M12. More sustained release of resveratrol was provided by sterilized CL M12, which had the same release rate as the counterpart M12 hydrogels. The reason behind the differences observed between sterilized and nonsterilized CLs is unclear since steam heat sterilization is the common technique for the sterilization of soft contact lenses and no relevant changes in the properties of the hydrogels were observed; for example, the swelling in water before sterilization was 107.4 ± 1.6% and after sterilization 100.6 ± 1.7%. A slight increase in the network density and reduction in pore size has been reported for HEMA-based sponges after autoclaving.^52^ A slight decrease in pore size might also decrease resveratrol release rate from the hydrogel bulk, which could explain that the initial amount released (resveratrol closer to the surface) was similar to both autoclaved and nonautoclaved hydrogels and that the differences in amount released became larger as the time proceeded. Resveratrol trapped in the bulk could find it more difficult to diffuse from the bulk toward the hydrogel surface from the more dense, sterilized CLs.

In any case, the CLs sustained the *in vitro* release for at least 1 day, and the amount of resveratrol released at 24 h was higher than for previously developed silicone (2.89 ± 0.58 μg/mg; 5.90 ± 0.63%) or HEMA-based (4.40 ± 0.18 μg/mg; 53.06 ± 4.77%) hydrogels with a 101 mM content in MPC.^24^ Overall, considering that resveratrol levels of 2.28 μg/mL protect retinal epithelial cells from UVA-induced oxidative damage (cell cultures)^53^ and from hyperglycemia-induced inflammation and gap junction intercellular communication degradation,^54^ the amount of resveratrol released would be enough for therapeutic effects.

A release study in the presence of BSA and lysozyme, two of the major proteins present in the tear fluid, was also carried out for sterilized CLs. The concentration of each protein was fixed to 2.68 mg/mL.^38^ There were no differences in the release patterns recorded in NaCl 0.9% without proteins (6.44 ± 0.57 μg/mg) and in the presence of lysozyme (6.74 ± 0.75 μg/mg) (Figure S7 in Supporting Information). The amount of resveratrol released decreased in the presence of BSA to 4.74 ± 1.65 μg/mg. This correlates with a previously published work, where the amount of moxifloxacin released from Proclear 1-day CLs also decreased in artificial tear fluid which included albumin in its composition.^55,56^

**Figure 5:**
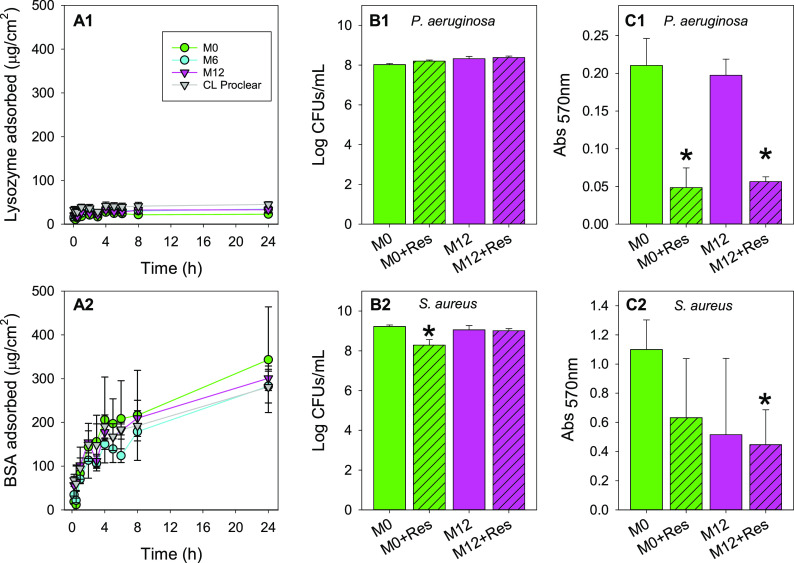
(A1, A2) Amounts of lysozyme and BSA adsorbed by the hydrogels when exposed to lysozyme (0.5 mg/mL, 6 mL) or BSA (2.68 mg/mL, 6 mL) solution in NaCl 0.9% and growth of (B1, C1) *Pseudomonas aeruginosa* and (B2, C2) *Staphylococcus aureus* biofilms on the surface of M0 and M12 hydrogels (without and with resveratrol) measured (B1, B2) as CFU counts and (C1, C2) through the MTT assay after 12 h of incubation (*n* = 3). Bacterial biofilms were grown with the novel hydrogel–agar interface method (Figure S1 in Supporting Information). *Statistically significant differences with respect to other groups (ANOVA, *p* < 0.05).

To gain an insight into the effects that proteins may have on the developed hydrogels, the sorption of lysozyme (pI = 11; 14.5 kDa) and BSA (pI = 4.7; 64.5 kDa) was monitored separately. Lysozyme had very low affinity for the hydrogels (Figure 5A1), while BSA was more prone to sorption (Figure 5A2). For comparison purposes, the results are shown as mass of protein sorbed per surface area of the hydrogels considering the two main surfaces of the disks (1 cm in diameter). As expected for pHEMA hydrogels,^57^ sorption of both proteins was quite low due to the moderate hydrophilic character of the network. BSA (anionic at physiological pH) was tested at much higher concentrations than in other reports, and it is known that because of its low conformational stability, BSA is prone to sorb onto any material irrespective of its surface properties.^58^ Differently, lysozyme and other proteins with high internal stability do not readily adsorb on hydrophilic networks. The larger size of BSA also causes this protein to deposit as coating layers on the pHEMA hydrogels, which may explain the delayed release recorded for resveratrol in medium containing BSA (Figure S7 in Supporting Information).

The incorporation of MPC had minor effects on protein adsorption which might be related to the fact that the monomer is well distributed in the bulk of the hydrogel and not only on the surface of the biomaterials as previously tested.^59^ There was a trend toward decrease of BSA sorption for hydrogels containing MPC, but the large standard deviations recorded prevented finding of statistically significant effects (Figure 5A2).

### Antibiofilm Properties

Next, blank and resveratrol-loaded M0 and M12 hydrogels were challenged regarding capability to inhibit biofilm formation of *P. aeruginosa* and *S. aureus*. The assay was performed using a novel disk–agar biofilm growth method in 24-well agar plates instead of the AAA-model which uses liquid culture medium,^24^ to resemble more closely the clinical conditions where the CL is placed on the ocular surface and not in a large volume of bacteria culture. In this case, the agar nourished the inoculum located above it and bacteria remained just below the CL. Biofilm formation was evaluated through the determination of CFUs/mL (Supporting Information) and the MTT assay. The MTT assay is an indirect highly sensitive method based on the conversion of the reagent MTT into formazan crystals by living cells which determines mitochondrial activity and not only biofilm mass.^60^

In the case of *P. aeruginosa* (Figure 5B1,C1), the CFUs method did not reveal differences among treatments, but the MTT assay evidenced a decrease in biofilm activity for both M0 and M12 hydrogels once loaded with resveratrol. This finding agrees well with previous reports on the activity of resveratrol against *P. aeruginosa*.^24^ In the case of *S. aureus* (Figure 5B2,C2), M0 hydrogels loaded with resveratrol showed a decrease in biofilm formation through the CFUs determination, and the MTT assay pointed to a decrease in the biofilm activity after the loading of resveratrol both for hydrogel compositions and for blank hydrogels with the highest content in MPC. Nevertheless, the experimental variability was quite high for *S. aureus*, and only resveratrol-loaded M12 hydrogels led to statistically significant differences with respect to the control M0 (ANOVA, *p* < 0.05).

### Anti-Inflammatory Activity

The anti-inflammatory activity of resveratrol solutions has been previously demonstrated both *in vitro* and *in vivo*(61) but not after loading in ophthalmic hydrogels. In the present study, the secretion of TNF-α and IL-6, two pro-inflammatory cytokines involved in different ocular diseases, was evaluated using LPS stimulated-macrophages. As shown in Figure S8, the production of both TNF-α and IL-6 significantly decreased for M0 and M12 hydrogels after being loaded with resveratrol (M0R and M12R), with values similar to those of negative control, demonstrating the capacity of the hydrogel disks to release therapeutically relevant amounts of resveratrol. Nonloaded M12 hydrogel disks also caused a decrease in the production of both cytokines, probably due to the presence of MPC.^62^ As expected, a decrease in TNF-α production was also observed for freshly prepared 50 μM resveratrol solutions, while even lower resveratrol concentrations (25 μM) attenuated IL-6 production. Overall, the obtained results highlight the effectiveness of resveratrol-loaded M0 and M12 hydrogels to preserve the anti-inflammatory capability of resveratrol and decrease the secretion of both proinflammatory cytokines.

### HET-CAM Test

The chorioallantoic membrane (CAM) of fertilized eggs has a vasculature comparable to the conjunctiva, and therefore, it is considered as an alternative to *in vivo* testing for evaluating the ocular compatibility of new formulations.^39^ After 5 min in contact with the CAM, no hemorrhage, vascular lysis, or coagulation was observed, so all hydrogel compositions could be considered as nonirritating (Figure S9 in Supporting Information). Differently, the NaOH solution used as positive control resulted in hemorrhage, vascular lysis, and coagulation showing an irritation score (IS) of 19.74.

### *In Vivo* Tests: Resveratrol Released and Accumulated in the Ocular Tissues

After the *in vitro* tests and the *in ov*o prescreening of ocular tolerability, an *in vivo* experiment using six male white New Zealand rabbits was performed to evaluate the capability of the developed CL M12 to release therapeutical amounts of resveratrol in the tear fluid and the capacity of resveratrol to pass through and accumulate into the different ocular tissues. New Zealand rabbit eyes have been extensively used in ophthalmology research due to their similarity with human eyes.^63^ The protocol chosen for the *in vivo* experiment was similar to one recently developed by some of us to test resveratrol-loaded Pluronic F127 micelle eye-drops, in terms of amount of resveratrol delivered to the eye (approximately 200 μg), sampling time, and sample processing.^41^ A solution of resveratrol in water could not be used as a reference due to its poor aqueous solubility. It should be noted that drug encapsulation in micelles increases the apparent solubility but also facilitates drug ocular tissue penetration compared to a free drug solution.^64,65^ Therefore, the performance of CL M12 was compared to the results obtained for an eye drop formulation that had already evidenced good outcomes.^41^

### Resveratrol Levels in Tear Fluid

**Figure 6:**
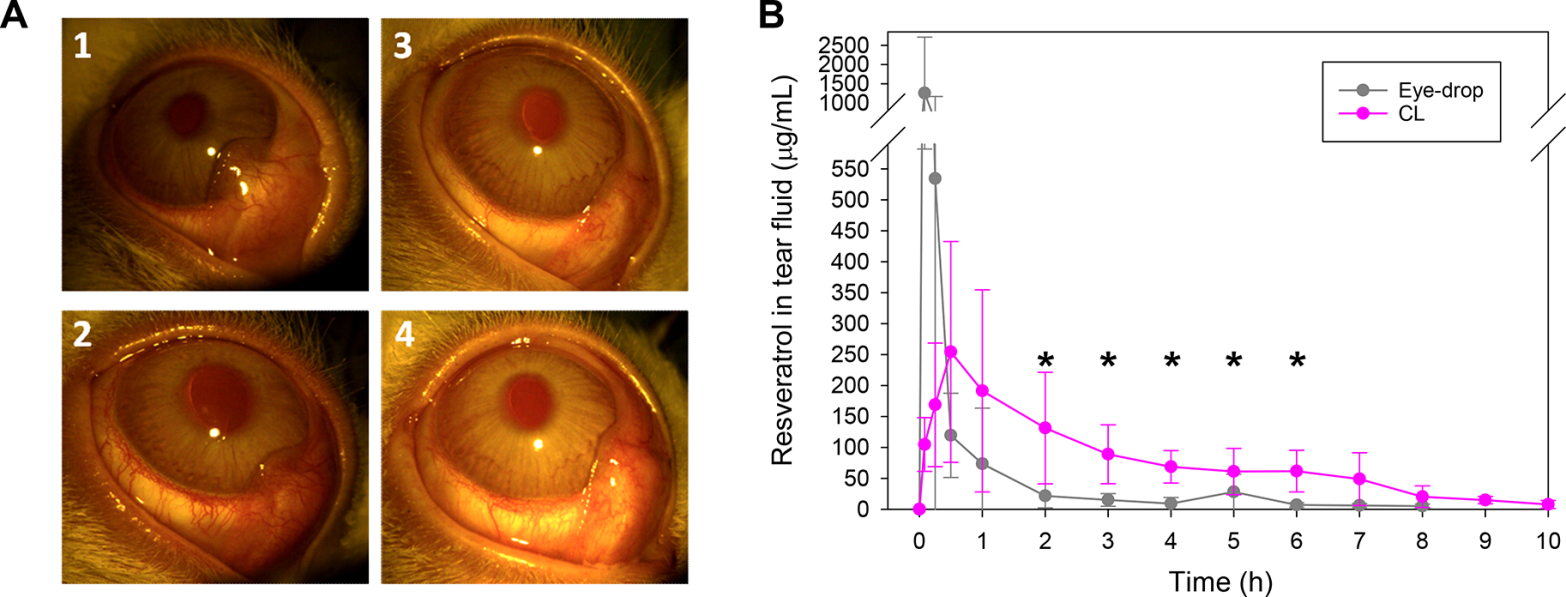
(A) Slit-lamp images of rabbit’s eyes (1, 3) before treatment and (2) after 8 h wear of the CLs and (4) 10 h wear of the CLs and (B) *in vivo* tear fluid levels of resveratrol recorded during wearing of CLs for 8 h (*n* = 6) and 10 h (*n* = 3) and compared with the values previously reported^43^ for instillation of one drop of resveratrol-loaded Pluronic F127 dispersion (50 μL, 4 mg/mL, *n* = 4). Shown are mean values and standard deviations. *Statistical differences (*p* < 0.05) between CL group and eye-drop group.

The slit-lamp images of the ocular surface of the rabbits collected, for both groups, before the beginning of the experiment and at the end demonstrated that CL M12 was well tolerated (Figure 6A). No signs of ocular irritation or damage were observed in any of the two CL groups. It should be noted that the CLs were directly taken from the loading solutions prepared with ethanol/water 10:90 v/v medium and simply rinsed with sterile saline solution before eye insertion. Thus, traces of the diluted ethanol solution did not compromise the ocular safety, and the CLs could be stored in the loading solution without risk of discharge, ready for use.

The *in vivo* release profiles of resveratrol in tear fluid are shown in Figure 6B. Typically, after one eye-drop instillation, the highest drug concentration is recorded in the first few minutes and then the level exponentially decreases.^64,65^ That was the case of the previously reported resveratrol-encapsulated micelles that provided a peak of resveratrol in the tear fluid at 5 min (1255.5 ± 456.0 μg/mL) and then the concentration rapidly decreased to 8.66 ± 10.40 μg/mL in the first 4 h.^41^ Differently, resveratrol-loaded CLs led to lower concentrations of resveratrol at the peak, but the release was sustained over a period of 8 h; the highest concentration was 254.3 ± 178.3 μg/mL at 0.5 h. Statistical analysis of resveratrol levels in tear fluid was carried out by comparing the data recorded during CLs wearing for 8 h (for all rabbits that wore CLs) with those previously reported for resveratrol-encapsulated micelles.^41^ ANOVA and multiple range test (*p* < 0.05) revealed that resveratrol-loaded CLs provided notably higher resveratrol levels, compared to the micelle dispersion, at time points 120, 180, 240, 300, and 360 min.

### Resveratrol Ocular Distribution

**Figure 7:**
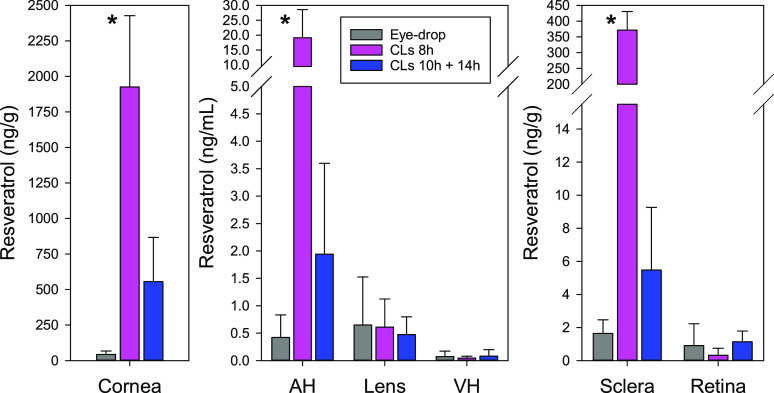
Levels of resveratrol in rabbit eye tissues recorded after 8 h wearing of resveratrol-loaded CLs (*n* = 3) and levels recorded 14 h after discontinuation of resveratrol-loaded CLs that were worn for 10 h (*n* = 3). The values were compared to those recorded after instillation of one drop of resveratrol-loaded micelle dispersion (50 μL, 4 mg/mL, *n* = 4) using the data reported in ref (43). The bars represent mean values and standard deviations. *Statistical difference found for CLs worn for 8 h compared to micelle dispersion and CLs worn for 10 h with a clearance period of 14 h (*p* < 0.05).

Biodistribution of resveratrol in the anterior and posterior ocular segments was also evaluated (Figure 7). Resveratrol was detected in cornea, aqueous humor, lens, vitreous humor, sclera, and retina after 8 h CLs wearing and also after 14 h discontinuation of 10 h wearing CLs.

Wearing of resveratrol-loaded CLs (approximately 200 μg dose) for 8 h provided significantly larger values in cornea, sclera, and aqueous humor (ANOVA, *p* < 0.05; multiple range test) compared to the wearing for 10 h followed by 14 h discontinuation and the instillation of resveratrol-loaded micelles (although the dose was the same). The 8 h wear CLs treatment group led to resveratrol levels that ranked in the order cornea (1925 ng/g; sd 502) > sclera (371 ng/g; sd 59) > aqueous humor (19.2 ng/mL; sd 9.4) > lens (0.61 ng/g; sd 0.51) > retina (0.31 ng/g; sd 0.43) > vitreous humor (0.045 ng/mL; sd 0.035). Interestingly after wearing of resveratrol-loaded CLs for 10 h and a subsequent clearance period of 14 h, significant levels of resveratrol were still detected in cornea (555 ng/g; sd 311), sclera (5.48 ng/g; sd 3.79), aqueous humor (1.94 ng/mL; sd 1.66), and retina (1.14 ng/g; 0.64). Thus, compared to the eye drops,^41^ the administration of the same dose of resveratrol in CL M12 allowed for more efficient ocular delivery regarding both sustained supply of resveratrol to the eye surface and access of resveratrol to anterior and posterior segment tissues.

The number of papers reporting on drug levels in tissues after CL wearing (rabbits) is still limited. Ofloxacin levels in ocular tissues were recorded after 1 h wearing HEMA-based corneal CLs loaded with 282 μg of ofloxacin and ranked cornea (∼60 μg/g) > aqueous humor (∼10 μg/mL) > sclera (∼2 μg/g).^66^ CLs loaded with timolol (277 μg) that were worn for 5 h led to higher levels in cornea (∼15 μg/g) followed by aqueous humor (∼6 μg/g) and sclera (∼3 μg/g).^67^ Thus, although the values recorded for resveratrol were lower, the tissue concentrations ranked in an order similar to the previous reports. The lower values recorded for resveratrol may be linked to the sampling time of 8 h, in which the CL is almost exhausted and clearance mechanisms may predominate. In this regard, resveratrol levels in eye tissues after 8 h wearing of CL M12 were higher than those recently reported for pravastatin sodium delivered from HEMA-based CL copolymerized with hydrophobic (ethylene glycol phenyl ether methacrylate) and amino-bearing (2-aminoethyl methacrylamide hydrochloride) monomers, which lead to pravastatin contents of 158.46 ± 31.8 ng/g in cornea and 1.53 ± 0.59 ng/g in sclera.^68^

### Antioxidant Activity of Resveratrol Worn CLs

A DPPH assay was performed to evaluate the capacity of CL M12 to preserve the antioxidant activity of the resveratrol remaining in the polymeric network after the *in vivo* experiment. This, in turn, could demonstrate the capacity of the CLs to protect resveratrol from light degradation under the conditions in which the *in vivo* test was performed. Resveratrol was extracted from the CLs using ethanol/water 50:50 v/v solution (extraction medium) and quantified to be 2.43 ± 0.99 μg (out of approximately 200 μg initially loaded) for CLs worn for 8 h and to be 0.57 ± 0.18 μg for CLs worn for 10 h. The antioxidant activity of the resveratrol-containing extraction medium was tested in parallel with that of freshly prepared extraction medium (used as control) to discard any effect of the medium itself. The antioxidant activity was expressed as μg/mL of DPPH in the medium and as percentage as previously described.^69^

Resveratrol extracted from the CL M12 that was worn for 8 h caused a remarkable decrease in the concentration of DPPH radicals, while no changes were recorded for the control (Table 3). This means that the designed CLs effectively protected resveratrol from its degradation and the small amount of resveratrol remnant in the CLs at the end of the 8 h *in vivo* test still preserved its antioxidant activity. A change of the color from purple to yellow with the consequent decrease in absorbance at 517 nm was observed due to the DPPH radical scavenging activity of the extracted resveratrol (Figure S10 in Supporting Information). CL M12 that was worn for 10 h was nearly exhausted, and the antioxidant activity was not measurable.

**Table 3:** DPPH Levels (μg/mL) and Scavenging Effect (%) of Resveratrol Extracted from CL M12 That Had Been Worn for 8 h (Concentration Tested, 0.76 ± 0.29 μg/mL) Compared to a Control Ethanol/Water 50:50 v/v Solution without Resveratrol^a^

CL	DPPH (μg/mL)	DPPH scavenging effect (%)
CL 8 h wear	3.50 ± 0.59	27.23 ± 11.46
control	4.90 ± 0.05	0

a All data are mean ± standard deviations (*n* = 3).

### *In Vitro*–*In Vivo* Correlations

Mimicking *in vitro* the conditions in which drug release occurs from a CL in the ocular surface has largely remained elusive due to the many factors that may exert an influence: tear fluid dynamic, tears volume and composition, blinking, ...^3^ Capability to predict *in vivo* behavior and quality assessment during production could strongly benefit from *in vitro* release tests that may provide biorelevant information. So far the few attempts to correlate *in vivo* and *in vitro* release from CLs pointed out that *in vitro* tests carried out with a small volume may be more predictive.^67,68,70^ In the case of resveratrol, a minimum volume of 6 mL of NaCl 0.9% was chosen due to its low aqueous solubility in order to prevent a rapid saturation of the release medium that could create artifacts in the release profile (as explained in the *in vitro* release section).

**Figure 8:**
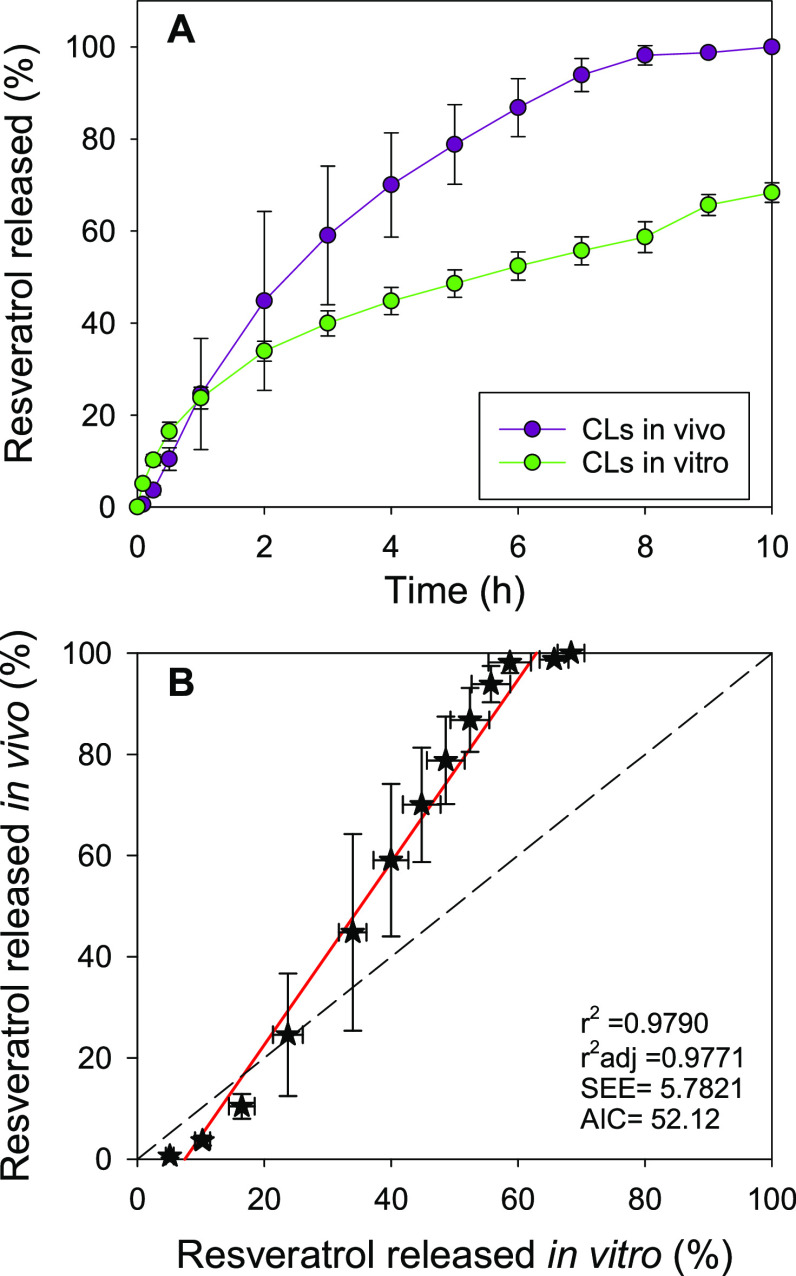
(A) *In vivo* resveratrol release profiles calculated from the levels in tear fluid provided by all CLs (*n* = 6 for time ≤8 h; *n* = 3 for time >8 h) and *in vitro* release profiles from sterilized CLs in NaCl 0.9% (*n* = 4). (B) Levy plot to estimate IVIVC. Ideal slope 1 is shown as reference with a dotted line. SEE: standard error of estimate. AIC: Akaike information criterion.

*In vivo* release profiles were constructed from the area under the curve of concentrations of resveratrol in tears versus time^68^ and then compared to the *in vitro* release profiles recorded in the absence of proteins (Figure 8). In the first 30 min of the release, the process was faster *in vitro* than *in vivo*, probably because of the much more volume available *in vitro* (compared to the few microliters available on eye surface) which created a more intense concentration gradient. Differently, after 1 h wearing the release *in vivo* became faster than *in vitro*, which may be due to components in the tears that favored the release and also to the continuous renovation of the tear layer.

The Levy plot (Figure 8B) showed a positive deviation with respect to slope 1; the experimental slope was 1.80. This means that after the initial 30 min, the *in vivo* release rate was almost double than that recorded *in vitro*. Similar patterns for the IVIVC have previously been reported for betaxolol hydrochloride when formulated in a film-embedded CL^70^ and for timolol and latanoprost formulated in CL containing the drug encapsulated in micelles.^43^ Nevertheless, the goodness of the fitting found for resveratrol was significantly better than for previously reported drug-loaded CLs.

## Conclusions

In the present work, the synthesis of HEMA-based hydrogels and CLs with high content in MPC (up to 381 mM or 12%) was successfully carried out in one step. MPC homogeneously distributed in the bulk of the network and increased its stiffness in the dry state but notably decreased Young’s modulus when wet. This softening together with an increase in solvent uptake and “free” water may notably enhance wearer comfort while preserving the required light transmission. Remarkably, although the CLs became more hydrophilic, the affinity for the hydrophobic antioxidant resveratrol was preserved and the strong interaction with the HEMA network allowed for the loading of therapeutically relevant amounts and the sustained release *in vitro*. Regarding the adsorption of tear proteins, lysozyme was not prone to deposit on the CLs, but the capability of MPC to hinder albumin deposition was not conclusive. Steam heat sterilized CLs provided more sustained release of resveratrol than the nonsterilized counterparts, which might be due to a small shrinking of the network with a decrease in mesh size. Antibiofilm studies revealed that combination of MPC and resveratrol aided in reducing the growth of *S. aureus* and *P. aeruginosa* on the hydrogel surface while exhibiting strong anti-inflammatory activity on macrophages. The developed CLs showed excellent ocular tolerance, and when compared with eye drops containing the same dose of resveratrol, their advantages in terms of sustained levels of resveratrol in tear fluid and in eye tissues (cornea, sclera, and aqueous humor) became evident. Moreover, the minor amount of resveratrol remaining in the CLs after 8 h of wearing still preserved its antioxidant activity, revealing the capability of the CLs to protect resveratrol from photodegradation. Finally, the Levy plot evidenced that after an initial lag time, *in vivo* release occurred faster than *in vitro* under the tested conditions. A strong correlation was found between the resveratrol percentages released *in vitro* and *in vivo*. In sum, CLs prepared with high proportion in MPC are pointed out as comfortable platforms for the sustained release of resveratrol that may help address ocular diseases that affect the anterior and posterior segments of the eye and that require antimicrobial, anti-inflammatory, and antioxidant management.

## Supporting Information Available

Additional experimental details of MTT assay and determination of colony-forming units (CFUs); schematic representation of the 24-well plate setup and hydrogel distribution for MTT and CFUs assay; UPLC chromatograms of resveratrol; solvent uptake (%) of hydrogel disks in water, SLF, and resveratrol loading solution before sterilization; light transmission recorded for hydrogel disks and CL M12 swollen in water, SLF, and resveratrol loading solution; resveratrol loading profile of nonsterilized CL M12 at 36 °C and 180 rpm for 24 h; resveratrol release profiles from nonsterilized and sterilized hydrogel disks in NaCl 0.9% and from sterilized CL M12 in NaCl 0.9% without and with incorporation of lysozyme and bovine serum albumin (BSA); anti-inflammatory activity of nonloaded and resveratrol-loaded M0 and M12 hydrogel disks and resveratrol solutions; images of the HET-CAM test; antioxidant activity of resveratrol extracted from CLs for 8 wearing compared to the control without resveratrol; tables contain the raw values of absorbances recorded during the loading and *in vitro* release experiments (PDF)
