# Supplementary material for: Phosphorylcholine-Based Contact Lenses for Sustained Release of Resveratrol: Design, Antioxidant and Antimicrobial Performances, and In Vivo Behavior
Source: ACS Appl Mater Interfaces. 2022 Dec 10;14(50):55431–46. doi: 10.1021/acsami.2c18217 (PMC9782386; doi:10.1021/acsami.2c18217)
Supplement: Supplementary file 1 — am2c18217_si_001.pdf [file am2c18217_si_001.pdf]

Phosphorylcholine-based contact lenses for sustained release of resveratrol: design, antioxidant and antimicrobial performances, and *in vivo* behavior

Maria Vivero-Lopez[1], Ana F. Pereira-da-Mota[1], Gonzalo Carracedo[2,3], Fernando Huete-Toral[2], Ana Parga[4], Ana Otero[4], Angel Concheiro[1] and Carmen Alvarez-Lorenzo[1,*]

[1]Departamento de Farmacología, Farmacia y Tecnología Farmacéutica, I+D Farma (GI-1645), Facultad de Farmacia, Instituto de Materiales (iMATUS) and Health Research Institute of Santiago de Compostela (IDIS), Universidade de Santiago de Compostela, 15782 Santiago de Compostela, Spain

[2]Ocupharm Research Group, Faculty of Optics and Optometry, Complutense University of Madrid, C/ Arcos del Jalon 118, 28037 Madrid, Spain

[3]Department of Optometry and Vision, Faculty of Optic and Optometry, Complutense University of Madrid, C/ Arcos del Jalon 118, 28037 Madrid, Spain

[4]Departamento de Microbiología y Parasitología, Facultad de Biología, Edificio CIBUS, Universidade de Santiago de Compostela, 15782 Santiago de Compostela, Spain

*Corresponding author. E-mail address: carmen.alvarez.lorenzo@usc.es

METHODS

MTT assay. The MTT assay was carried out as previously reported with slight modifications.[1,2] After 12 h of incubation, the hydrogels were rinsed in a 24-well plate filled with 2 mL of PBS pH 6.5 per well to remove bacteria that were not attached to the hydrogels surface and individually placed in sterile tubes with 1.8 mL of PBS pH 6.5 to be sonicated for 10 min to detach the formed biofilm. Then, 200 μL of the MTT solution (5 mg/mL) were added to each tube and incubated at 37 ˚C for 30 min under dark conditions. After incubation, the half of the MTT-containing PBS medium (1 mL) was removed from the tubes and replaced with the same volume of acid isopropanol (5% v/v 1 M HCl in isopropanol). The tubes were vortexed for 30 s and the absorbance of the medium (1 mL) measured at 570 nm (UV-Vis spectrophotometer Thermo Scientific Helios Omega). PBS medium treated in the same way was used as blank.

Determination of Colony-Forming Units (CFUs). The hydrogels were rinsed with PBS pH 6.5 as described above, placed into sterile tubes with 2 mL of the corresponding culture medium, and sonicated for 10 min followed by 30 s vortex to detach the biofilm bacteria. Then, bacteria detachment samples were serial diluted 1:10 by adding 0.5 mL of the bacteria sample to 4.5 mL of LB or TSB-1 medium for *P.*

aeruginosa and *S. aureus*, respectively. The 10^{-4} - 10^{-6} dilutions (100 µL) were seeded onto pre-dried TSA-1 or LB plates by the spread-plating procedure until agar absorbed the liquid and no droplets were observed. Agar plates were incubated at 37 ˚C for 24 hours. After incubation, bacteria colonies were counted and number of CFUs/mL in the original sample was calculated as follows:

$$\frac{CFUs}{mL} = \frac{n^{\underline{o}}\ of\ colonies\ \times dilution\ factor}{volume\ inoculated\ on\ culture\ plate\ (mL)} \qquad \text{Eq. (1)}$$

References

(1) Vivero-Lopez, M.; Muras, A.; Silva, D.; Serro, A. P.; Otero, A.; Concheiro, A.; Alvarez-Lorenzo, C. Resveratrol-Loaded Hydrogel Contact Lenses with Antioxidant and Antibiofilm Performance. *Pharmaceutics* **2021**, *13*(4), 532.

(2) Vivero-Lopez, M.; Xu, X.; Muras, A.; Otero, A.; Concheiro, A.; Gaisford, S.; Basit, A.W.; Alvarez-Lorenzo, C.; Goyanes, A. Anti-Biofilm Multi Drug-Loaded 3D Printed Hearing Aids. *Mater. Sci. Eng. C* **2021**, 119, 111606.

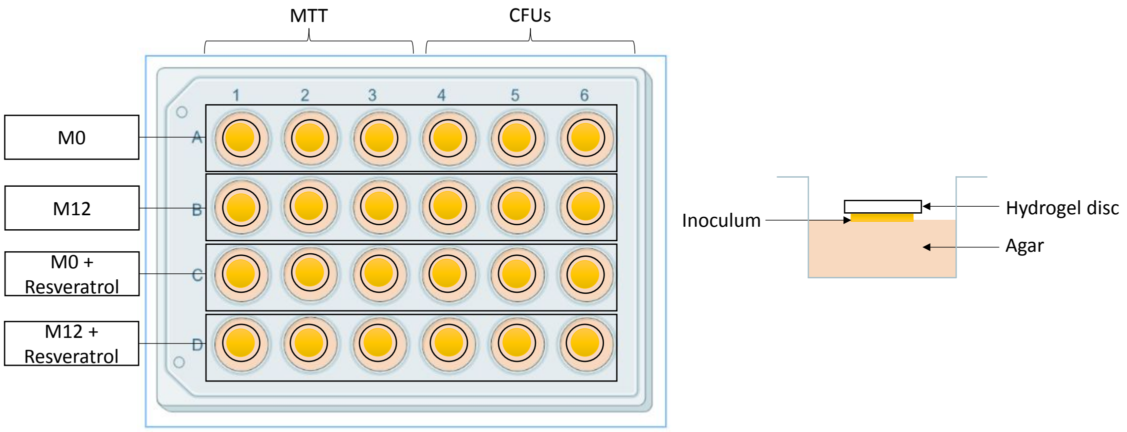

Figure S1. Schematic representation of the hydrogel disc-agar interface method for biofilm growth assessment. The 24-well plate setup and hydrogel distribution for MTT and CFUs assays are shown. Wells were inoculated with 20 µL of bacterial inoculum. Hydrogel codes as in Table 1.

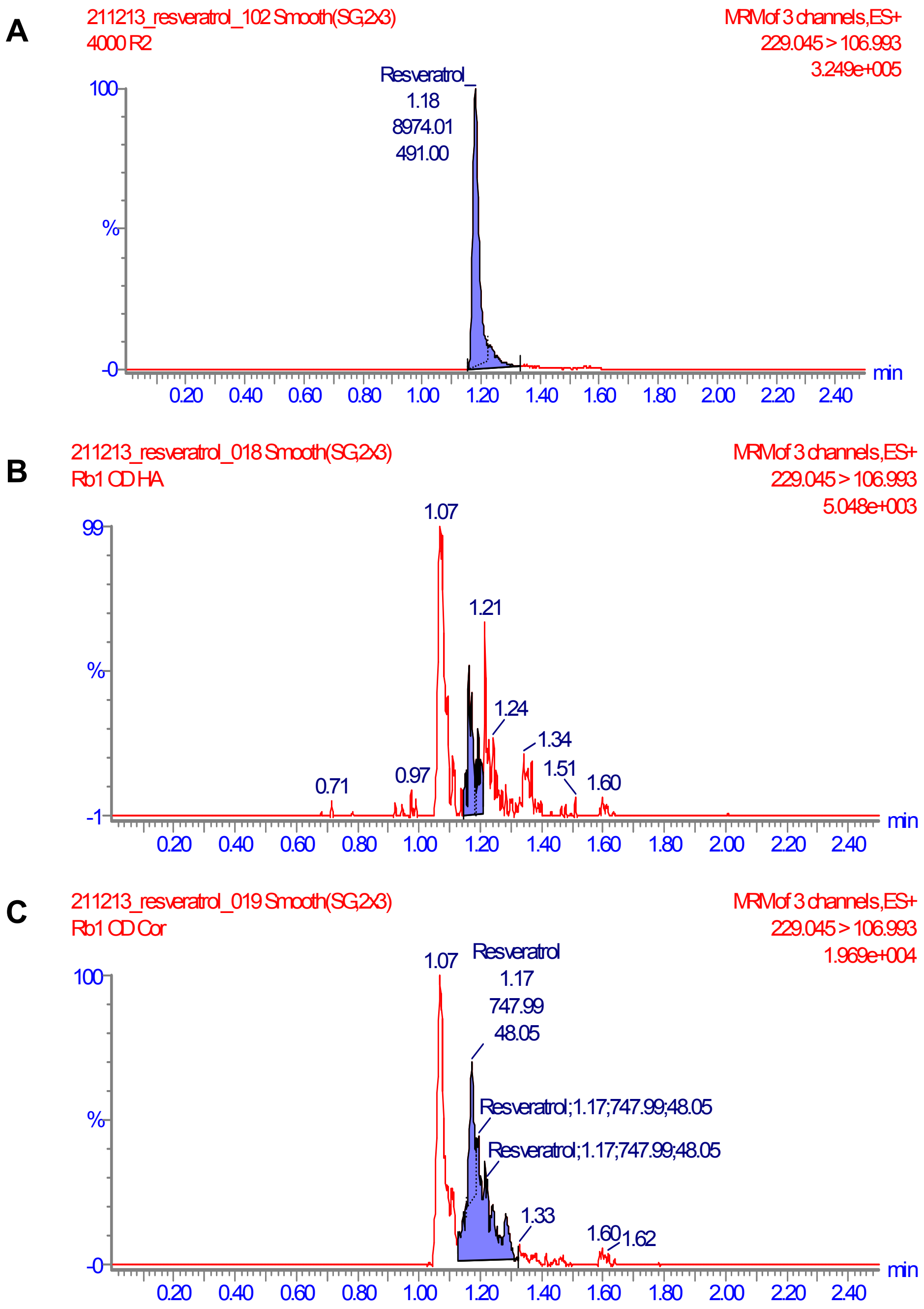

Figure S2. UPLC chromatogram of (A) a resveratrol standard (4000 ng/mL) prepared in the same medium as the samples, (B) resveratrol extracted from the aqueous humour of the right eye of rabbit 1, and (C) resveratrol extracted from the cornea of the right eye of rabbit 1.

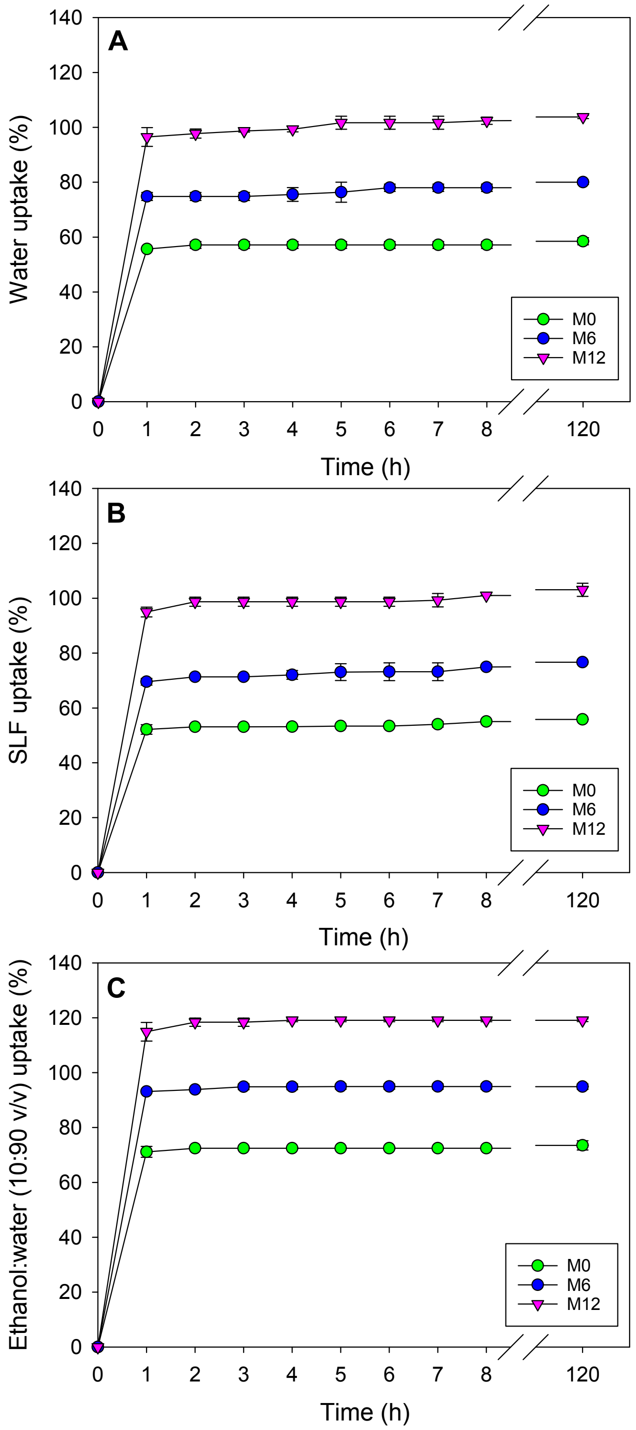

Figure S3. Solvent uptake (%) of hydrogel discs in (A) water, (B) SLF and (C) resveratrol loading solution before sterilization. Hydrogel codes as in Table 1.

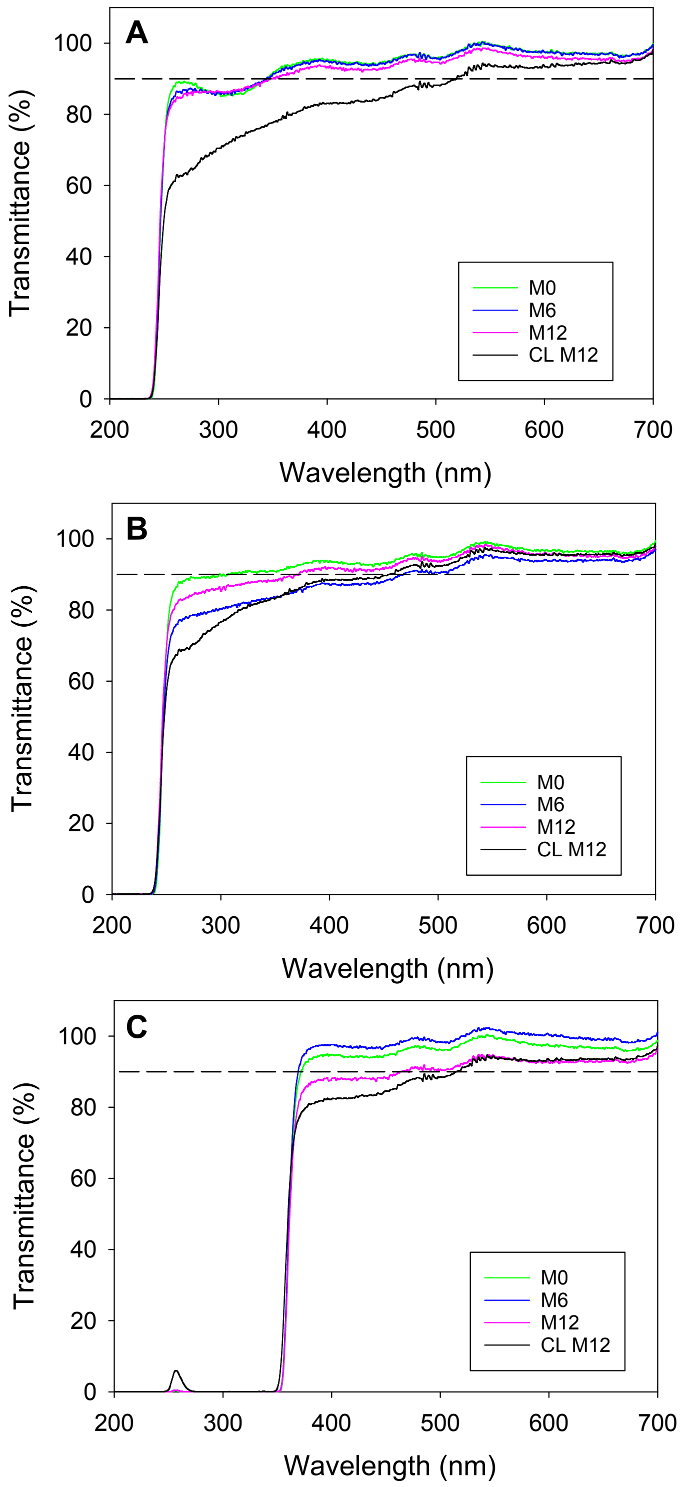

Figure S4. Light transmission recorded for hydrogel discs and CL M12 swollen in (A) water, (B) SLF and (C) resveratrol loading solution. Dashed lines indicate the required 90% transmittance value. Hydrogel codes as in Table 1.

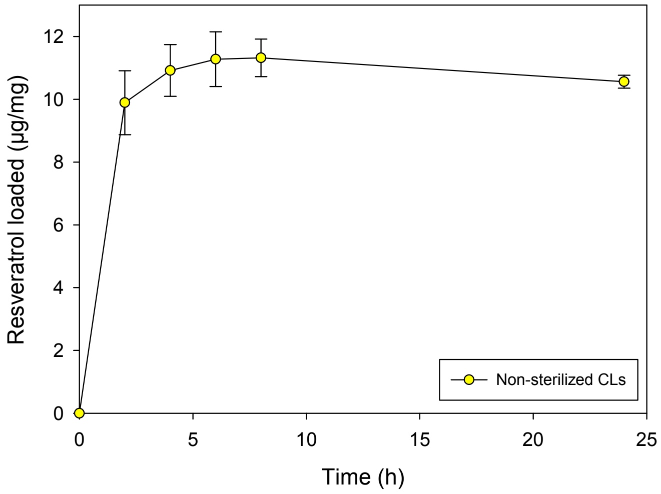

Figure S5. Resveratrol loading profile of non-sterilized CL M12 at 36 °C and 180 rpm for 24 h.

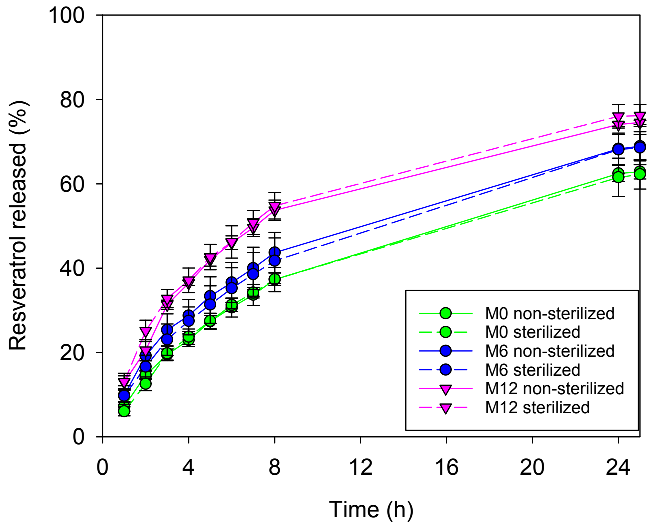

Figure S6. Resveratrol release profiles in NaCl 0.9% protected from light at 36 °C and 180 rpm from non-sterilized and sterilized hydrogel discs. Mean values and standard deviation (n=4). Codes as in Table 1.

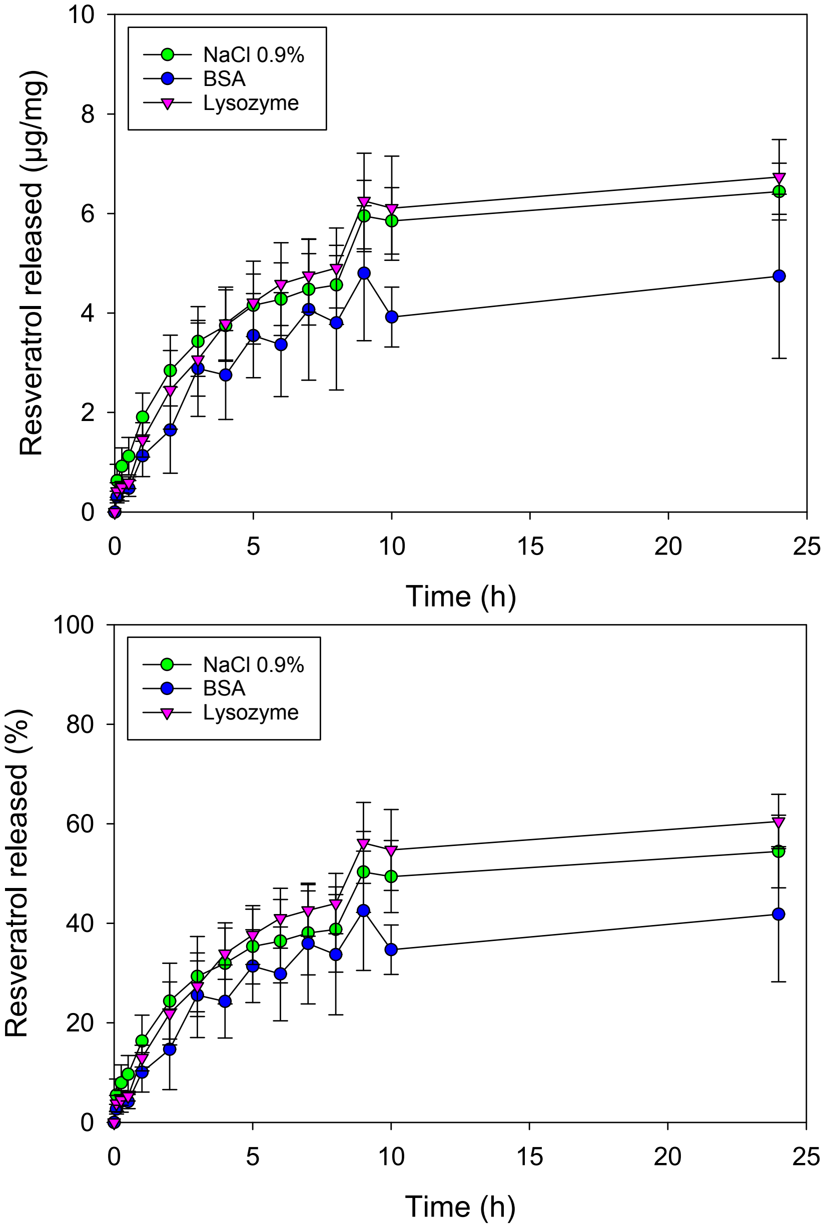

Figure S7. Resveratrol release profiles from sterilized CL M12 in NaCl 0.9% without and with incorporation of lysozyme and bovine serum albumin (BSA) (n = 4; mean values and standard deviations).

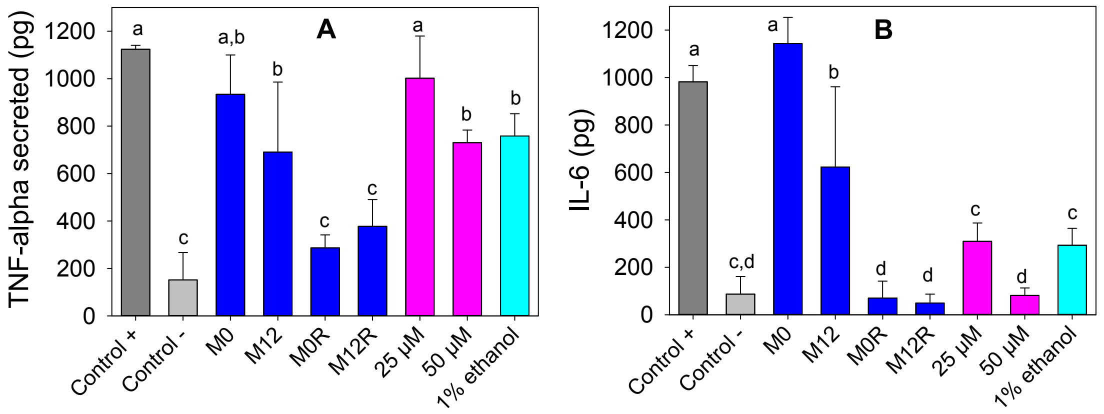

Figure S8. Effect of non-loaded and resveratrol-loaded M0 and M12 hydrogel discs and resveratrol solutions (25 and 50 µM) on the secretion levels of (A) TNF-α and (B) IL-6 from previously treated (hydrogels or resveratrol solutions) and stimulated (with LPS) macrophages. LPS-stimulated and non-stimulated cells acted as positive (Control+) and negative (Control-) controls, respectively. The bars represent mean values and standard deviations (n = 3). Statistical analysis: equal letters denoted homogeneous groups; different letters denoted statistically significant differences (ANOVA; multiple range test, $p < 0.005$).

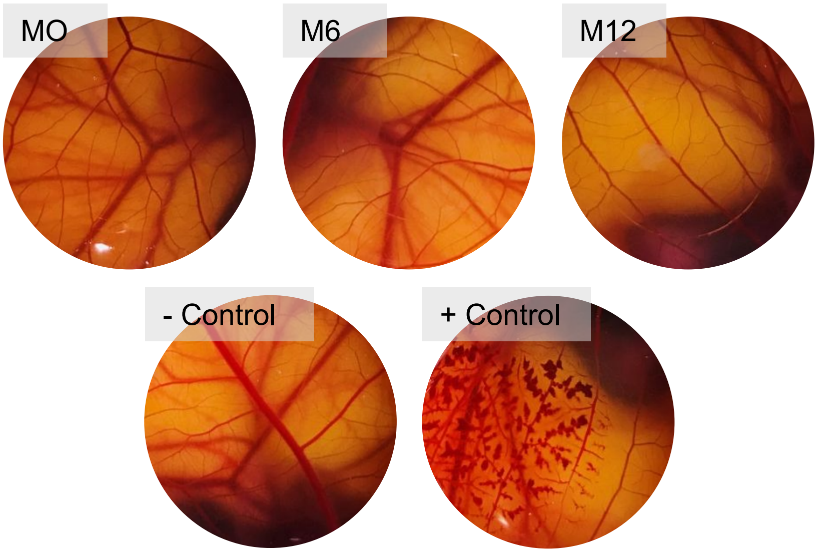

Figure S9. Images of the HET-CAM test. Codes as in Table 1.

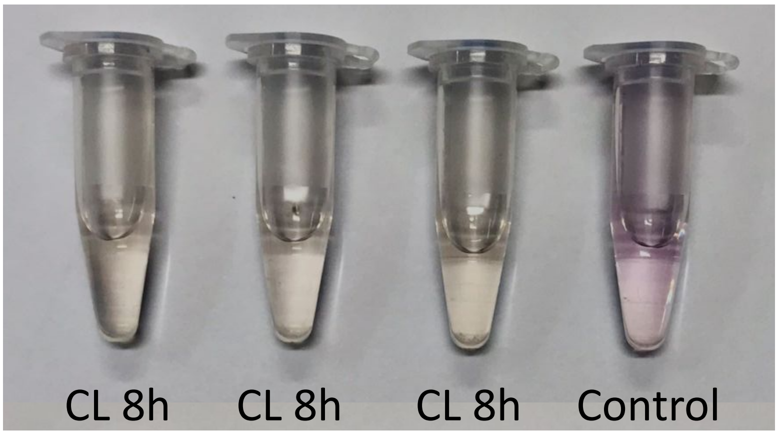

Figure S10. Antioxidant activity of resveratrol extracted from CLs after for 8 wearing (non-colored solutions) compared to the control (ethanol:water 50:50 v/v) without resveratrol (pale pink color).

Table S1. Absorbance values recorded during the loading experiment for both non-sterilized and sterilized (steam heat 121 °C, 20 min) hydrogel discs (n= 4). Hydrogel codes as in Table 1. The absorbance of the loading medium was measured every 2 h for the first 8 h and then each 24 h at 305 nm after leveling the aliquots (250 µL for the first 8 h and 500 µL after) to 5 mL with ethanol:water 10:90 v/v.

Hydrogel	Absorbance (305 nm)						
	2 h	4 h	6 h	8 h	24 h	48 h	72 h
M0 rep1	0.51998	0.46204	0.41196	0.43346	0.89345	0.93592	0.95793
M0 rep2	0.53123	0.46427	0.41356	0.44318	0.89759	0.95425	0.94144
M0 rep3	0.54509	0.48186	0.44396	0.46794	0.90717	0.94775	0.94973
M0 rep4	0.53984	0.46676	0.43561	0.46335	0.91131	0.96131	0.94170
M6 rep1	0.54922	0.47287	0.45273	0.45364	0.94169	0.94323	0.95158
M6 rep2	0.51163	0.44236	0.41870	0.46943	0.93223	0.97222	0.95891
M6 rep3	0.52690	0.43624	0.43122	0.47251	0.96019	0.98708	0.95707
M6 rep4	0.52282	0.44219	0.42718	0.47266	0.91913	0.98046	0.93955
M12 rep1	0.52590	0.47311	0.46945	0.46181	0.96770	1.00590	1.02120
M12 rep2	0.50812	0.45546	0.47147	0.47382	1.00120	1.00290	1.02240
M12 rep3	0.51029	0.47499	0.47860	0.48488	1.00910	1.00430	1.02680
M12 rep4	0.54379	0.48446	0.47523	0.47290	0.99987	1.01230	1.02390
M0 SH rep1	0.52682	0.46865	0.42114	0.39351	0.85127	0.89901	0.89603
M0 SH rep2	0.54619	0.46833	0.44640	0.44102	0.87397	0.93233	0.91840
M0 SH rep3	0.54552	0.48344	0.45106	0.44352	0.87262	0.94799	0.93307
M0 SH rep4	0.53040	0.45976	0.44878	0.43433	0.85180	0.94543	0.93264
M6 SH rep1	0.55709	0.46228	0.45222	0.43440	0.91208	0.96177	0.97073
M6 SH rep2	0.52254	0.46127	0.40282	0.43730	0.94443	0.98334	0.96956
M6 SH rep3	0.54528	0.47026	0.46536	0.44538	0.95582	0.99469	0.98214
M6 SH rep4	0.53878	0.42311	0.46128	0.38632	0.96011	0.99191	0.98771
M12 SH rep1	0.52130	0.46431	0.43555	0.44487	0.92610	1.01600	1.00960
M12 SH rep2	0.52658	0.47444	0.44621	0.46609	0.95886	1.00680	0.99714
M12 SH rep3	0.53818	0.47972	0.44688	0.47168	0.96704	1.00930	1.03700
M12 SH rep4	0.54645	0.48596	0.44774	0.46610	0.97234	1.04670	1.01120

Table S2. Absorbance values recorded during the release experiment for both non-sterilized and sterilized (steam heat 121 °C, 20 min) hydrogel discs (n=4). Hydrogel codes as in Table 1. The release was carried out in 6 mL NaCl 0.9% protected from light at 36 °C and 180 rpm. The absorbance of the release medium was measured at 305 nm (UV-Vis spectrophotometer Agilent 8534, Waldbronn, Germany) by taking aliquots of 3 mL at 1 and 2 h that were immediately returned to the Falcon® tubes after the measurement. After that, aliquots of 1 mL were taken at preestablished times, which were replaced with the same volume of NaCl 0.9% fresh solution. After measurement of the absorbance at 8 h, 6 mL more of NaCl 0.9% fresh solution were added increasing the release medium in all tubes to 12 mL. After measurement of the absorbance at 25 h, the hydrogels discs were transferred to falcon tubes containing 6 mL of fresh release medium and the test proceeded as explained above.

Hydrogel	Absorbance (305 nm)										
	1 h	2 h	3 h	4 h	5 h	6 h	7 h	8 h	24 h	25 h	32 h
M0 rep1	0.38836	0.74014	0.46757	0.47109	0.50202	0.46700	0.46612	0.47918	0.51690	0.47820	0.57547
M0 rep2	0.27537	0.63781	0.43809	0.42968	0.44656	0.44805	0.43260	0.43344	0.50668	0.47312	0.60083
M0 rep3	0.37788	0.71700	0.51187	0.50893	0.53529	0.52898	0.51144	0.50764	0.51576	0.47451	0.51138
M0 rep4	0.34108	0.71496	0.45433	0.47896	0.51641	0.53950	0.52830	0.54369	0.48370	0.44796	0.58972
M6 rep1	0.31156	0.61693	0.42271	0.42427	0.43219	0.42451	0.41471	0.44142	0.46313	0.43608	0.59176
M6 rep2	0.42478	0.82999	0.56259	0.55963	0.60601	0.57026	0.55191	0.55819	0.47562	0.44113	0.37430
M6 rep3	0.53868	0.89928	0.58586	0.56053	0.56635	0.55598	0.52637	0.50593	0.47935	0.44378	0.46990
M6 rep4	0.42877	0.97287	0.61676	0.57202	0.55532	0.52831	0.52975	0.50210	0.47804	0.44262	0.52141
M12 rep1	0.51287	0.88518	0.63877	0.59786	0.59240	0.57205	0.53814	0.52088	0.43018	0.39926	0.34257
M12 rep2	0.48777	0.66666	0.58970	0.63191	0.63979	0.60856	0.58410	0.57551	0.42690	0.39523	0.36830
M12 rep3	0.43964	0.80437	0.61592	0.61080	0.64260	0.62015	0.59489	0.58032	0.41924	0.38864	0.36525
M12 rep4	0.58372	0.85548	0.60577	0.60235	0.59284	0.57076	0.53695	0.52538	0.43527	0.40423	0.37793
M0 SH rep1	0.25008	0.61620	0.44783	0.46897	0.48307	0.51096	0.51200	0.49938	0.45983	0.45164	0.50108
M0 SH rep2	0.30107	0.50895	0.50331	0.50191	0.51375	0.52292	0.50605	0.50353	0.50728	0.46521	0.46895
M0 SH rep3	0.26601	0.64396	0.45788	0.48350	0.50092	0.49724	0.48613	0.48286	0.50486	0.46554	0.43007
M0 SH rep4	0.35023	0.66553	0.50759	0.52114	0.51100	0.51229	0.48405	0.47731	0.51300	0.47256	0.56698
M6 SH rep1	0.32770	0.59041	0.40393	0.43060	0.42903	0.43207	0.41804	0.42189	0.48576	0.45469	0.54542
M6 SH rep2	0.45018	0.74659	0.47578	0.51493	0.47337	0.47163	0.46924	0.44622	0.48476	0.45110	0.50947
M6 SH rep3	0.41899	0.65641	0.51271	0.52473	0.54312	0.53943	0.53561	0.50119	0.46623	0.43483	0.48644
M6 SH rep4	0.46262	0.85027	0.57395	0.54707	0.56446	0.56083	0.52992	0.53118	0.48248	0.43829	0.45735
M12 SH rep1	0.59706	1.01950	0.65891	0.63109	0.63142	0.59398	0.55182	0.52994	0.41960	0.38254	0.33463
M12 SH rep2	0.51453	0.94460	0.59563	0.58190	0.60251	0.55113	0.55886	0.53618	0.41948	0.38668	0.34618
M12 SH rep3	0.43942	0.96535	0.61028	0.60366	0.59896	0.58683	0.56063	0.54476	0.40683	0.36989	0.33456
M12 SH rep4	0.39501	0.80305	0.56977	0.53081	0.53315	0.50433	0.53405	0.52013	0.42894	0.40150	0.37266
